# New insights into optimal storage conditions for raw Pu-erh tea: modulating post-fermentation flavor via microbial metabolism under controlled water activity

**DOI:** 10.1016/j.jare.2025.10.078

**Published:** 2025-11-05

**Authors:** Bingsong Ma, Longjie Xu, Shuang Liang, Shuya Han, Cunqiang Ma, Xinghui Li, Yongquan Xu, Yue Shi, Junfeng Yin

**Affiliations:** a Tea Research Institute Chinese Academy of Agricultural Sciences, Key Laboratory of Tea Biology and Resources Utilization, Ministry of Agriculture, 9 South Meiling Road, Hangzhou 310008, China; b College of Horticulture, Nanjing Agricultural University, Nanjing 210095 Jiangsu, China

**Keywords:** Raw Pu-erh tea, Water activity, Microbial metabolism, Kokumi, Storage flavor, Post-fermentation

## Abstract

**Introduction:**

The post-fermentation storage of raw Pu-erh tea (RPT) is a key process that contributes to the development of its characteristic aged flavor. Microbial activity plays a vital role in this transformation which strongly influenced by water activity (A*w*). However, its specific effects on microbial community dynamics and associated metabolic contributions during RPT storage remain unclear.

**Objectives:**

This study aims to elucidate the mechanisms by which different A*w* levels shape microbial communities and drive flavor compound formation during RPT storage.

**Methods:**

RPT samples were stored under defined A*w* conditions, during which microbial communities were characterized using Internal Transcribed Spacer (ITS) sequencing, and flavor metabolites were analyzed through gas chromatography-mass spectrometry (GC–MS), liquid chromatography-mass spectrometry (LC-MS). Correlation analyses and core strain-inoculated fermentations under simulated and realistic A*w* conditions were conducted to investigate the relationship between microbial dynamics and flavor evolution.

**Results:**

Controlled A*w* conditions significantly influenced the microbial composition and flavor chemistry of RPT. A*w* above 0.60 enriched fungal genera such as *Papiliotrema*, *Hannaella*, *Toxicocladosporium*, and *Pestalotiopsis*, which were strongly correlated with the accumulation of aging-associated terpenoids (e.g., *α*-terpineol, terpinen-4-ol, cedrol, linalool) and kokumi-active compounds including flavoalkaloids and amino acid derivatives. A core yeast isolate, *Papiliotrema flavescens*, exhibited metabolic activity at A*w* > 0.60 under both simulated and realistic storage conditions. It produced *β*-glucosidase, which promoted the hydrolysis of glycosidic precursors and the accumulation of linalool and methyl salicylate, confirming its role in microbial-driven flavor development. However, A*w* levels exceeding 0.70 promoted the formation of undesirable volatiles like *N*,*N*-dimethyl-propanamide, toluene, and isophorone, indicating potential quality deterioration.

**Conclusion:**

A*w* is a pivotal parameter that connects storage safety with microbial-driven flavor development in RPT. An optimal range of 0.60–0.70 promotes beneficial transformations while minimizing off-odor risks. These findings highlight A*w* regulation as a practical and novel strategy for improving aging quality.

## Highlights

• Water activity (A*w*) defines microbial shifts and flavor metabolite formation during RPT storage.
• *Papiliotrema* and *Hannaella* enhance the accumulation of aging-aroma terpenoids in higher-A*w* RPT.
• Elevated A*w* levels promote kokumi-active flavoalkaloids through fungal metabolism.
• Microbial metabolism of *Papiliotrema flavescens* RPT is verified at A*w* above 0.60.
• A*w* range of 0.60 – 0.70 optimally balances flavor development and microbial safety.

## Graphical abstract

**Figure:**
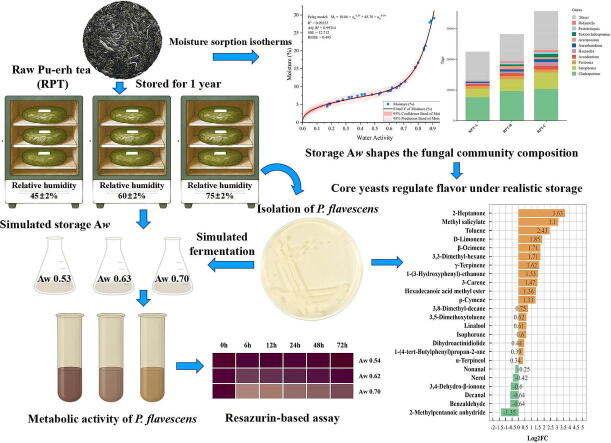

## Introduction

Tea is among the most widely consumed beverages worldwide, characterized by a remarkable diversity of varieties and flavor profiles that result from its broad geographic origins and diverse processing techniques [1,2]. While green teas are traditionally valued for their freshness, others such as Pu-erh tea are renowned for improving in quality with aging [3]. In particular, raw Pu-erh tea (RPT), a post-fermented tea produced from sun-dried green tea (crude Pu-erh tea, CPT) of large-leaf tea species grown in Yunnan, China, is often described as “the aged, the better,” with long-term natural storage developing kokumi (thickness, mouthfulness, and continuity) taste and unique aging aroma [4,5]. These sensory attributes also increase the market value of RPT increase with aging, and well-preserved products fetch premium prices in both domestic and international markets [6].

Storage is increasingly recognized as an extension of tea processing, wherein heat, moisture, oxygen, and light continuously drive the transformation of intrinsic compounds, actively shaping tea quality rather than merely preserving it [7]. Variations in temperature and humidity during storage can markedly alter microbial community structures and metabolic profile, leading to diverse chemical and sensory outcomes. Comparative studies have demonstrated that wet-hot storage conditions accelerate the degradation of flavan-3-ols, amino acids, and flavonol glycosides, while promoting the formation of flavoalkaloids and other polyphenol-derived metabolites that enhance sensory quality [8,9]. In contrast, dry-cold conditions induce slower transformations, requiring extended storage durations to develop comparable mellow and smooth mouthfeel characteristics. Additionally, environmental differences affect the volatile profiles, with wet-hot conditions favoring the formation of aged and woody aroma compounds, while dry-cold conditions tend to preserve fresh and floral notes [10]. However, the specific microbial contributions to these transformations during tea storage remain poorly understood and appear to be highly influenced by environmental conditions.

Recent studies have demonstrated that chemical transformations during tea storage are not solely governed by abiotic factors, but are also strongly influenced by microbial metabolism, which plays a pivotal role in shaping tea quality [11]. For instance, the “jinhua” (golden-flower) fungus *Eurotium repens*, which develops during natural aging, has been shown to dramatically alter volatile organic compound profiles, catalyze flavonoid conversions, and reduce bitterness and astringency while enhancing floral, woody, and mellow notes in both ripened Pu-erh tea (RiPT, produced from CPT by moisture-assisted pile-fermentation) and white teas [12]. Similarly, during white tea storage, core bacterial genera such as *Pantoea* and *Massilia*, along with fungal genera including *Aspergillus* and *Cladosporium*, have been linked to the degradation of glycosylated flavonoids, the accumulation of flavonoid aglycones, and the decline of free amino acids, likely due to microbial utilization and conversion into flavor-active metabolites [13]. Moreover, key “core” taxa of stored dark teas was identified by microbial community analysis, such as *Aspergillus*, *Gluconobacter*, *Pantoea*, and *Talaromyces* that contributed to the transformation of polyphenols and other key compounds, promoting reactions such as flavonoid methylation and glycosylation and thereby facilitating the progressive development of flavor and aroma [[14], [15], [16]]. Overall, tea processing altered the chemical composition of tea leaves, which subsequently leads to the selective enrichment of specific microbial communities and reshaping of core taxa, ultimately influencing the development of post-fermentation flavor [17,18]. Evidence from naturally aged RPT also supports that microbial community succession contributes to aroma differentiation during long-term storage, while recent advances in pile-fermented Pu-erh tea have further clarified the close linkage between microbial metabolism and flavor compound transformation, suggesting that microorganisms under different moisture conditions can drive flavor-active metabolite conversions [19,20]. This process is likely mediated by microbial secreted enzymes that link microbial metabolism with the conversion of tea flavor precursors [21]. However, storage conditions, particularly the availability of water, also play a critical role in shaping microbial structure and activity, yet their specific effects remain insufficiently elucidated [22].

Water activity (A*w*), as a thermodynamic measure of biologically available water, is a central determinant of food stability, directly influencing microbial viability, enzyme activity, and the rates of non-enzymatic reactions such as lipid oxidation and Maillard-type browning [22,23]. Compared with total moisture content, A*w* provides a more accurate and reliable indicator of microbial metabolic potential and chemical reactivity, and is thus critical for maintaining the stability, quality, and safety of tea during storage [24,25]. In Pu-erh tea storage, *Aspergillus flavus* (*A. flavus*) posed a risk of aflatoxin contamination under improper humidity control, though its growth was largely inhibited at A*w* levels below 0.80 [26]. Apart from temperature influence, most *Aspergillus* species fail to grow at A*w* levels below 0.76–0.87 [24,27,28]. However, excessive humidity, such as relative humidity (RH) above 88 %, can shift microbial community composition toward spoilage-associated taxa, leading to off-flavors and reduced sensory quality [29]. In contrast, for RPT that rely on slow post-fermentation, excessively low A*w* resulting from overly desiccated storage may suppress microbial metabolism and delay flavor maturation [29]. Microbial metabolic activity can occur even in A*w* ranges insufficient to support proliferation, offering a potential strategy to promote flavor development while minimizing spoilage risk [30]. Moreover, foodborne microorganisms exposed to environmental stresses may remain in sublethal states with residual metabolic activity and recover rapidly once favorable conditions are restored [31]. These findings highlight the possibility of an optimal A*w* window that enables beneficial microbial activity without promoting microbial overgrowth, thereby minimizing the risk of spoilage while allowing beneficial biochemical transformations.

In summary, although the importance of A*w* and microbial metabolism in tea aging is well recognized, a clear knowledge gap remains in elucidating their controlled impact on flavor chemistry. Few studies have quantitatively assessed how defined A*w* levels affect microbial succession, metabolic activity, and the formation of flavor-active compounds during the storage of RPT. In this study, a controlled storage system was established under constant temperature with three defined A*w* levels. Microbial community dynamics were characterized using internal transcribed spacer (ITS) sequencing, while flavor-targeted metabolomics was conducted through headspace solid-phase microextraction coupled with gas chromatography–mass spectrometry (HS-SPME-GC–MS) and ultra-high-performance liquid chromatography coupled with Q-exactive Orbitrap mass spectrometry (UHPLC-Q-Exactive/MS). In addition, microbial strains isolated from stored RPT were subjected to simulated A*w* conditions to assess their metabolic activity and potential pathways involved in flavor modulation under storage safety A*w*. This integrated approach provides new insights into optimizing post-fermentation storage through A*w* control, enabling flavor enhancement while ensuring microbial safety.

## Materials and methods

***Material and reagents***.

***Sample collection***

RPT was prepared from one bud with two or three leaves harvested in spring from Mangzhi Tea Mountain, located in Xishuangbanna, Yunnan Province. Fresh leaves were subjected to enzymes inactivation, rolling and sun-drying to produce CPT, which was subsequently steamed and compressed into RPT. The samples were stored in a temperature-controlled tea warehouse (25 ± 2 °C), where three sealed professional humidity-control cabinets regulated the microenvironment. These enabled stable one-year storage under different conditions: A (45 ± 2 % RH), B (60 ± 2 % RH), and C (75 ± 2 % RH), and designated as RPT-A, RPT-B, and RPT-C, respectively. Following storage, the samples were collected, and their moisture content and A*w* were measured. All samples were stored at −80 °C until further analysis.

***Reagents***.

Formic acid, trifluoroacetic acid, methanol, and acetonitrile (LC-MS grade) were purchased from CNW Technologies GmbH (Bielefeld, North Rhine-Westphalia, Germany). Ethanoic acid (LC-MS grade) was obtained from Thermo Fisher Scientific (Shanghai, China). Ethanol (≥ 99.5 %, purity) was purchased from Sinopharm Chemical Reagent Co., Ltd. (Shanghai, China). Ethyl decanoate (≥ 98 %) was obtained from Aladdin (Shanghai, China). A standard n-alkane mixture (C5–C25), used for retention index (RI) calculation in GC–MS analysis, was obtained from ANPEL Laboratory Technologies Inc. (Shanghai, China).

The TSINGKE Plant DNA Extraction Kit, TSE102 Gold Mix (2 × ), ITS1 (5′-TCCGTAGGTGAACCTGCGG-3′) and ITS4 (5′-TCCTCCGCTTATTGATATGC-3′) primers (10  μM), high-purity low-EEO agarose, DL5000 DNA Marker, and pre-heated TE buffer (65 °C, pH 8.0) were all obtained from Tsingke Biotechnology (Beijing, China). Taq DNA polymerase, dNTP Mix, and 6 × DNA loading buffer were also purchased from Tsingke. DNA electrophoresis was performed using 1.2 % agarose gel in 1 × TAE buffer prepared from analytical-grade reagents.

For microbial cultivation and metabolic assays, Potato Dextrose Agar (PDA) and Potato Dextrose Broth (PDB) were obtained from ACMEC (Shanghai, China). Glycerol (≥ 99.0 %), sodium chloride (NaCl, ≥ 99.5 %), and sucrose (≥ 99.5 %) were obtained from Macklin Biochemical Co., Ltd. (Shanghai, China) and used to adjust water activity in culture media. Resazurin (RZ, > 90 %) used for assessing microbial metabolic activity, was purchased from Mairida (Beijing, China). Fluorescent enzyme activity assay kits for peroxidase (POD) and polyphenol oxidase (PPO) were purchased from Shanghai Yuanye Bio-Technology Co., Ltd. (Shanghai, China). The *β*-glucosidase (*β*-GC) assay kit was obtained from Shanghai Acmec Biochemical Technology Co., Ltd. (Shanghai, China).

***Moisture sorption isotherms of RPT***.

The A*w* of RPT samples was adjusted and measured following a modified protocol based on Zhao et al. [26]. Briefly, 50 g of RPT was initially dried at 60 °C to reduce its A*w* to approximately 0.20. The dried sample was then placed in a sealed glass desiccator containing a natural sponge at the bottom. To gradually increase A*w*, 0.5–2.0 mL of distilled water was incrementally added to the sponge. The desiccator was maintained at 25 °C for one week to allow the sample to reach moisture equilibrium. After equilibration, A*w* was measured using an A*w* meter (WA-160A, Meter Environmental Protection Instrument Co., Beijing, China), and moisture content was determined using a halogen moisture analyzer (VM-E10, VicoMeter Instruments, Jiangsu, China).

Moisture sorption isotherm data were modeled using seven nonlinear equations commonly applied in tea and food research: Peleg, Guggenheim–Anderson–de Boer (GAB), Brunauer–Emmett–Teller (BET), Oswin, Henderson, Halsey, and Smith models [32,33]. All models were fitted to the experimental data using the nonlinear least squares method in Origin 2024b (OriginLab, Northampton, MA, USA). Model performance was evaluated based on the coefficient of determination (R^2^), adjusted R^2^ (Adj. R^2^), root mean square error (RMSE), and sum of squared errors (SSE).

***Microbiome analysis by ITS sequencing***.

Fungal community composition was assessed by targeting the internal transcribed spacer 1 (ITS1) region. Total DNA was extracted from tea samples using the MinkaGene Plant DNA Kit (mChip BioTech, Guangzhou, China) according to the manufacturer’s protocol. DNA concentration and integrity were assessed using an Agilent 5400 system (Agilent Technologies, Santa Clara, CA, USA). Each experimental group included three independent biological replicates. Detailed procedures for PCR amplification, library preparation, Illumina sequencing, and bioinformatic processing are provided in the Supplementary Materials.

***Detection of RPT metabolites stored at different Aw***.

***Volatile metabolites (VMs) analysis by HS-SPME-GC–MS***.

The VMs were extracted using HS-SPME. Briefly, 0.1  g of ground tea sample (40 mesh) was placed in a 20  mL headspace vial, followed by the addition of 5  mL of boiling distilled water and 20  μL of ethyl decanoate (5 μg/L, internal standard). The vial was incubated in a 60 °C water bath for 5 min. Subsequently, a preconditioned SPME fiber was exposed to the headspace at 60 °C for 60  min. The adsorbed volatiles were then thermally desorbed in the GC injector at 230 °C for 5 min [34]. Each experimental group included three independent biological replicates. The detailed procedures for gas chromatographic parameters, mass spectrometer settings, and data processing are provided in the Supplementary Materials.

***Non-volatile metabolites (NVMs) analysis by UHPLC-Q-Exactive/MS***.

For NVMs analysis, 0.1  g of ground tea sample (40 mesh) was extracted with 10  mL of 70 % methanol containing 0.1 % trifluoroacetic acid in a 70 °C water bath for 30 min. The extract was centrifuged at 8000 × g for 10 min at 4 °C, and the resulting supernatant was filtered through a 0.22 μm nylon membrane. All filtrates were transferred into glass vials and stored at 4 °C prior to analysis. Quality control (QC) samples were prepared by pooling equal aliquots from each sample and were analyzed intermittently throughout the sequence to monitor system stability and data quality [35]. Each experimental group included three independent biological replicates.

***Isolation and identification of core fungal strain***

Microorganisms present in the stored RPT samples were isolated, purified, and identified. Briefly, 25 g of tea sample was added to 225  mL of sterile water, thoroughly shaken, and serially diluted to obtain a 1:100 working suspension. A 10 μL aliquot of the diluted suspension was inoculated onto PDA plates using a sterile loop and incubated at 28 °C for 5  days. Distinct colonies were sub-cultured repeatedly to obtain purified isolates [36]. Detailed procedures for genomic DNA extraction, ITS region amplification, and taxonomic identification of purified isolates are provided in the Supplementary Materials.

***Resazurin-based assessment of microbial metabolic activity under simulated storage Aw***.

To simulate the A*w* conditions of RPT storage, different concentrations of glycerol, NaCl, and sucrose were added to PDB to adjust A*w* levels [37]. Specifically, the PDB-A condition contained 4.5 M glycerol, 1.5 M NaCl, and 1.8  M sucrose; PDB-B contained 4.0  M glycerol, 1.0  M NaCl, and 1.5 M sucrose; and PDB-C contained 4.0 M glycerol, 1.0 M NaCl, and 1.0 M sucrose. These concentrations were designed to approximate A*w* of 0.54, 0.62, and 0.70, respectively, thereby mimicking the storage conditions of RPT-A, RPT-B, and RPT-C.

The inoculum was prepared at a concentration of 5 × 10⁶ cells/mL, and 5  mL was added to 100  mL of each medium. Cultures were incubated at 30 °C with shaking at 200  rpm. Samples were collected at six time points: 0, 6, 12, 24, 48, and 72  h. At each time point, 200  μL of RZ solution (10  mM) was added to 10  mL of culture broth. Following addition, the mixtures were further incubated, and metabolic activity was assessed based on the extent of color change.

Colorimetric changes were quantified using a color difference meter (Konica Minolta CM-5, Konica Minolta, Japan), and L*, a*, and b* values were recorded. The total color difference (ΔE) was calculated to reflect the degree of resazurin reduction, with higher ΔE values indicating greater microbial metabolic activity [38,39].

***Fermentation of tea infusion by RPT-derived core strain under storage simulated storage Aw***.

Take 3 g of CPT and infuse it with 150 mL of boiling water in a 200 mL Erlenmeyer flask, then incubate in a 100 °C water bath for 15 min. Filter the infusion leaves, and prepare 100  mL of PDB-A, PDB-B, or PDB-C medium with 10 mL of the resulting tea infusion replacing distilled water, according to the formulation described in the above section, to obtain tea infusion fermentation samples TA, TB, and TC. All cultures were incubated on a rotary shaker at 30 °C and 200 rpm. Fermentation samples were collected at 0, 1, 2, 3, and 7 days. At each time point, 1  mL of culture broth was withdrawn, diluted to 10 mL with methanol, centrifuged at 8000 × g for 10 min at 4 °C, and filtered through a 0.22 μm nylon membrane. Tea infusion metabolites were analyzed using the UHPLC-Q-Exactive/MS platform with consistent chromatographic parameters and data processing methods across all samples. All experiments were performed with three independent biological replicates. Metabolite data were normalized by *Z*-score transformation, and time-resolved regression analyses were conducted using Pearson correlation with corresponding *p*-values.

***Storage of tea inoculated with RPT-derived core strain under realistic condition***

Take 18  g of CPT (initial moisture content 7.0 ± 0.1 %) and place it into tissue culture bottles, which are sterilized at 121 °C for 15 min under standard autoclaving conditions. 1 mL of the suspension (5 × 10⁶ cells/mL) was added to the treatment group (PF), and an equal volume of sterile distilled water was added to the non-inoculated control group (NPF). The moisture content was adjusted to 12.0 ± 0.3 %, and the bottles were stored in an incubator at 25 °C and 70 % RH for 10 months. After storage, enzymatic activities were measured as described in the Supplementary Materials. VMs was analyzed following the same protocols used for RPT samples. All experiments were conducted in triplicate using independent biological replicates.

### Statistical analysis

Data are expressed as mean values with standard deviations (SD). Statistical analyses, including the independent-sample *t*-test, one-way analysis of variance (ANOVA) with Tukey’s honestly significant difference (HSD) test were conducted using SPSS 20.0 (Armonk, NY, USA) to determine significance levels, while data visualization was performed using Origin 2024b (OriginLab, Northampton, MA, USA). For metabolites in the TA, TB, and TC groups, data were normalized by *Z*-score transformation across all time points within each compound, and temporal dynamics were analyzed by linear regression with fermentation time as the predictor, while Pearson correlation coefficients (*r*) with corresponding *p*-values were calculated to determine the significance and direction of time-dependent trends. Differential metabolite selection was based on VIP > 1 combined with false discovery rate (FDR)-adjusted *q* < 0.05 to control for multiple testing. Partial least squares discriminant analysis (PLS-DA), Venn diagrams, Spearman correlation analysis, and inter-omics chord diagrams were generated using the Metware Cloud platform (https://cloud.metware.cn).

## Results and discussion

***Moisture sorption isotherms of stored RPT***.

The hygroscopic behavior of tea varies considerably depending on variety and processing methods, leading to distinct A*w* values at the same moisture content [22,40]. Due to water serves as a critical medium for both chemical reactions and microbial activity, A*w* is widely recognized as a more reliable indicator of tea storage conditions than relative humidity or moisture content, particularly in evaluating microbial risks and storage stability during post-processing [23,41]. By establishing moisture sorption isotherms for RPT, A*w* can be effectively employed to guide storage safety and improve product quality throughout storage. The isotherm of tea typically exhibits a Type II sigmoid profile, which reflects multilayer adsorption behavior. Several mathematical models such as Peleg, GAB, BET, Oswin, Halsey, Henderson, and Smith have been successfully applied to describe moisture sorption in green, black, and dark teas, providing reliable estimates across a wide range of A*w* conditions [22,33,42,43].

**Fig. 1:**
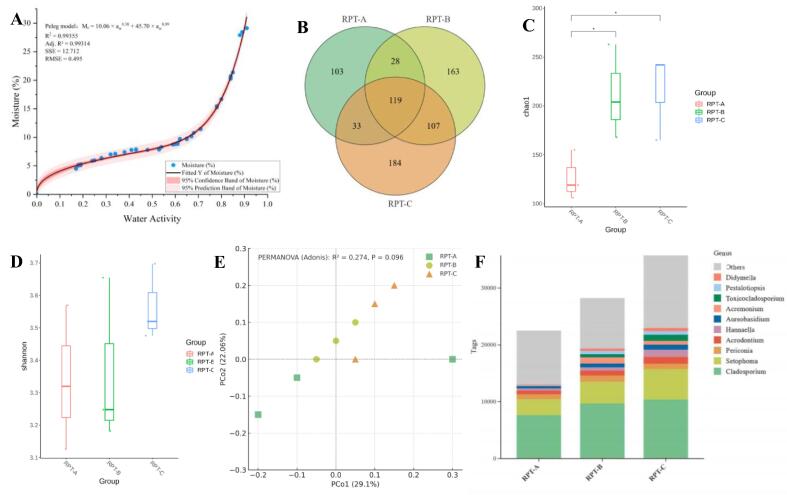
**Effects of water activity (A*w*) on moisture sorption behavior and fungal community diversity in stored raw Pu-erh tea (RPT).** (A) Moisture sorption isotherm of RPT fitted using the Peleg model. (B) Venn diagram of shared and unique ASVs among RPT samples under different A*w* conditions. (C) Chao1 richness index and (D) Shannon diversity index representing alpha diversity. (E) Principal coordinates analysis (PCoA) based on Bray–Curtis dissimilarity representing beta diversity. (F) Relative abundance of dominant fungal genera under different A*w* conditions.

Comparative fitting parameters of all isotherm models are presented in Table S1. In particular, the Peleg model (Fig. 1A) demonstrated the best overall performance, with the highest coefficient of determination (R^2^ = 0.9936) and the lowest RMSE (0.495). This finding is consistent with previous studies, in which the Peleg model demonstrated excellent goodness of fit across a wide temperature range (20–50 °C) for various tea types and forms, including black tea, green tea powder, and granules [32,33]. To assess storage safety, the Peleg model was used to estimate the critical A*w* corresponding to the maximum moisture content allowed by the national standard (≤ 13 %, GB/T 22,111 – 2008). The calculated value was 0.741, which is lower than the minimum threshold required for the growth of common spoilage fungi such as *Aspergillus niger* and *Aspergillus flavus* [26,27], thereby confirming the microbiological safety of RPT stored under this moisture limit. Notably, the actual A*w* values of the three stored RPT samples were 0.53 (RPT-A), 0.61 (RPT-B), and 0.71 (RPT-C), respectively, with replicate variations falling within the 0.01 precision of the water activity meter, confirming reproducibility under stable storage conditions. All values were below the safety thresholds predicted by the model, thereby confirming microbiological safety under typical storage conditions [32].

***Fungal composition of stored RPT with different Aw***.

Storage conditions significantly affect the microbial community, influencing the the metabolism and transformation of chemical constituents. To investigate fungal composition under different A*w* conditions, ITS sequencing was used to analyze the fungal community structure in RPT.

**Fig. 2:**
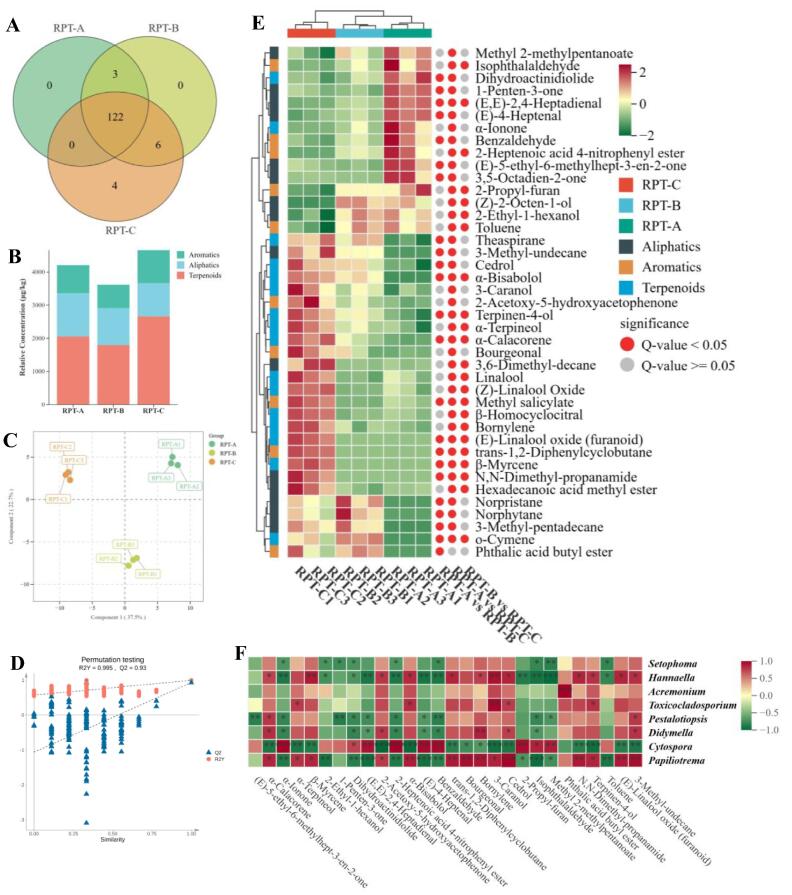
Changes in volatile metabolites (VMs) of raw Pu-erh tea (RPT) stored under different water activity (A*w*) levels. (A) Venn diagram showing the number of shared and unique VMs detected under three A*w* levels. (B) Relative concentrations of total VMs under three A*w* levels. (C) Partial least squares discriminant analysis (PLS-DA) score plot and (D) Permutation test showing clear separation of three storage A*w* based on VMs. (E) Differential VMs (VIP > 1and FDR-adjusted *q* < 0.05) associated with storage A*w* and aroma characteristics. (F) Spearman correlations between fungal genera and VMs (|*ρ*| > 0.85).

The Venn diagram showed both common and unique fungal ASVs across the three A*w* conditions (Fig. 2B). A total of 119 ASVs were common to all samples, represented a stable core microbiota. However, RPT-C contained the highest number of unique ASVs (184), followed by RPT-B (163) and RPT-A (103), indicating that higher A*w* supports a more diverse fungal community. Additionally, RPT-B and RPT-C shared 107 ASVs not found in RPT-A, while RPT-A shared only 28 and 33 ASVs with RPT-B and RPT-C, respectively. These results suggest that higher A*w* increases fungal diversity and promotes community similarity under moderately and highly humid conditions.

The Chao1 richness index increased significantly (*p* = 0.018 for RPT-A vs RPT-B; *p* = 0.024 for RPT-A vs RPT-C) with rising A*w* levels (Fig. 1C), with RPT-A showing the lowest and RPT-C the highest fungal richness. Similarly, the Shannon diversity index showed an increasing trend from RPT-A to RPT-C, indicating enhanced community evenness under high A*w* levels (Fig. 1D). Although the differences in Shannon values were not statistically significant, the consistent upward trend together with Chao1 results suggests that A*w* may influence both fungal richness and overall diversity during RPT storage. Principal coordinates analysis (PCoA) based on Bray–Curtis dissimilarity revealed distinct clustering of fungal communities according to A*w* levels (Fig. 1E), and PERMANOVA confirmed that A*w* explained 27.4 % of the variation in community composition (R^2^ = 0.274, *p* = 0.096). RPT-A separated distinctly from RPT-B and RPT-C, which were more closely clustered, demonstrating that increased water availability induces substantial shifts in fungal community composition. The first two axes (PCo1 and PCo2) explained 29.10 % and 22.06 % of the total variance, respectively, reinforcing that A*w* is a key environmental driver shaping fungal assemblages during storage.

*Ascomycota* predominated in all storage conditions, followed by *Basidiomycota* at the phyla level, consistent with findings in stored white and Pu-erh teas, with both phyla showing significantly higher relative abundance at increased A*w* levels (*p* < 0.05, Fig. S1) [11]. At the genus level, *Cladosporium* and *Setophoma* consistently represented the major components, with their abundance progressively increasing from RPT-A to RPT-C (Fig. 1F). Within the genera of moderate abundance, *Hannaella* (*p* = 0.0357), *Papiliotrema* (*p* = 0.0400), and *Didymella* (*p* = 0.0419) exhibited significant increases under higher A*w* conditions, whereas *Cytospora* (*p* = 0.00061) decreased significantly with rising A*w*, showing the highest abundance in RPT-A. Other genera such as *Periconia*, *Acrodontium*, and *Toxicocladosporium* displayed upward trends without statistical significance (Table S2). These dominant genera contrasted sharply with those commonly found in RiPT, where *Aspergillus* and other thermotolerant fungi prevail, indicating that the low-A*w*-driven transformation in RPT as a distinct post-fermentation trajectory compared to the high-A*w*-driven pile-fermentation of RiPT [20]. Overall, these results demonstrate that increasing A*w* promotes both the richness and abundance of key fungal taxa, while selectively inhibiting others such as *Cytospora*, highlighting the role of A*w* as a critical environmental filter that shapes fungal succession and influences metabolite profiles during RPT storage.

***Effects of Aw on microbial community shifts and VM Profiles***.

***VMs composition of stored RPT with different Aw***.

A total of 134 VMs were identified by GC–MS from RPT samples stored under different conditions, which were categorized into 3 major classes (Table. S3), with terpenoids (34 compounds) predominating, followed by aliphatics (62),and aromatics (38). As shown in Fig. 2A, a total of 122 VMs were consistently detected across all three storage conditions, indicating that the core volatile profile remained stable during short-term storage. In contrast, 2-heptenoic acid 4-nitrophenyl ester, (*Z*)-2-octen-1-ol, and 2-ethyl-2-methyl-tridecanol were exclusively detected in RPT-A and RPT-B, while six compounds, such as *α*-bisabolol and octadecane, were only found in RPT-B and RPT-C. Four VMs such as *β*-myrcene, *trans*-1,2-diphenylcyclobutane, *N*,*N*-dimethyl-propanamide, and (*E*)-linalool oxide (furanoid) were exclusively detected in RPT-C, suggesting that higher A*w* may promote oxidative reactions and microbial metabolism [12,44,45].

A*w* significantly affected the distribution and relative concentration of volatile compound classes in stored RPT (Fig. 2B). The highest total volatile content was observed under high A*w* conditions (RPT-C, 4660.1 ± 506.6 μg/kg), followed by RPT-A (4207.3 ± 662.2 μg/kg), with RPT-B showing lowest values (Table S3). Aliphatics, including aldehydes, alcohols, and ketones associated with green and fresh aromas, were most abundant in RPT-A under low A*w* conditions. In contrast, aromatics and terpenoids showed the highest relative concentrations under high A*w*, but exhibited the lowest levels under moderate A*w*, resembling the pattern of total volatile concentration. Specifically, aromatics were significantly higher in RPT-C (991.6 ± 127.3 μg/kg) than in RPT-B (*p* = 0.044), while RPT-A showed moderate levels (843.3 ± 132.1 μg/kg). Terpenoid concentrations increased from 2054.5 ± 318.1 μg/kg in RPT-A and 1797.1 ± 147.9 μg/kg in RPT-B to 2661.8 ± 266.4 μg/kg in RPT-C, with a significant difference observed between RPT-B and RPT-C (*p* = 0.014) and a near-significant difference between RPT-A and RPT-C (*p* = 0.060), possibly due to carotenoid degradation or glycoside hydrolysis promoted by higher moisture levels [46].

The PLS-DA model revealed clear separation among RPT-A, RPT-B, and RPT-C based on their VMs profiles, indicating that storage A*w* significantly influenced aroma composition (Fig. 2C). The model showed excellent fit and predictive performance, as confirmed by permutation testing (R^2^Y = 0.995, Q^2^ = 0.930, *p* < 0.005) (Fig. 2D). A total of 41 VMs were selected as discriminant compounds, defined as metabolites contributing significantly to the separation among A*w* groups, based on model importance (VIP > 1.0) combined with FDR-adjusted significance (*q* < 0.05) (Fig. 2E). Notably, several VMs associated with fresh, green, and slightly floral aromas, such as (*E*,*E*)-2,4-heptadienal, (*E*)-4-heptenal, 1-penten-3-one, and dihydroactinidiolide, showed significantly higher abundance in the lowest-A*w* condition (RPT-A) [47]. These short-chain aldehydes and ketones, derived from lipid oxidation, were key contributors to grassy and green aromas in tea [48]. In contrast, several aging-related aroma compounds, including linalool, its oxides [(*Z*)- and (*E*)-linalool oxide], cedrol, theaspirane, *α*-bisabolol, and hexadecanoic acid methyl ester, were consistently detected across all storage conditions but showed significantly higher concentrations under increased A*w* (Fig. 2E). This result suggested that elevated A*w* may promote a faster accumulation of aging-associated volatiles during RPT storage. In particular, linalool, (*Z*)-linalool oxide, and (*E*)-linalool oxide (pyranoid) showed significant (*q* < 0.05) to highly significant (*q* < 0.01) increases under higher A*w*, exhibiting the highest levels in RPT-C. Previous studies have reported that fungi such as *Eurotium cristatum* and yeasts including *Saccharomyces cerevisiae*, commonly found in post-fermented teas, produced enzymes such as *β*-glucosidase, to liberate glycosylated linalool precursors [12,49,50]. This pattern suggests that elevated A*w* may facilitate both enzymatic oxidative processes and microbial transformations, particularly enhancing the biosynthesis of linalool-derived VMs, one of the main contributors to the aging aroma in Pu-erh tea.

***Impact of fungal communities on VMs during RPT Storage***.

**Fig. 3:**
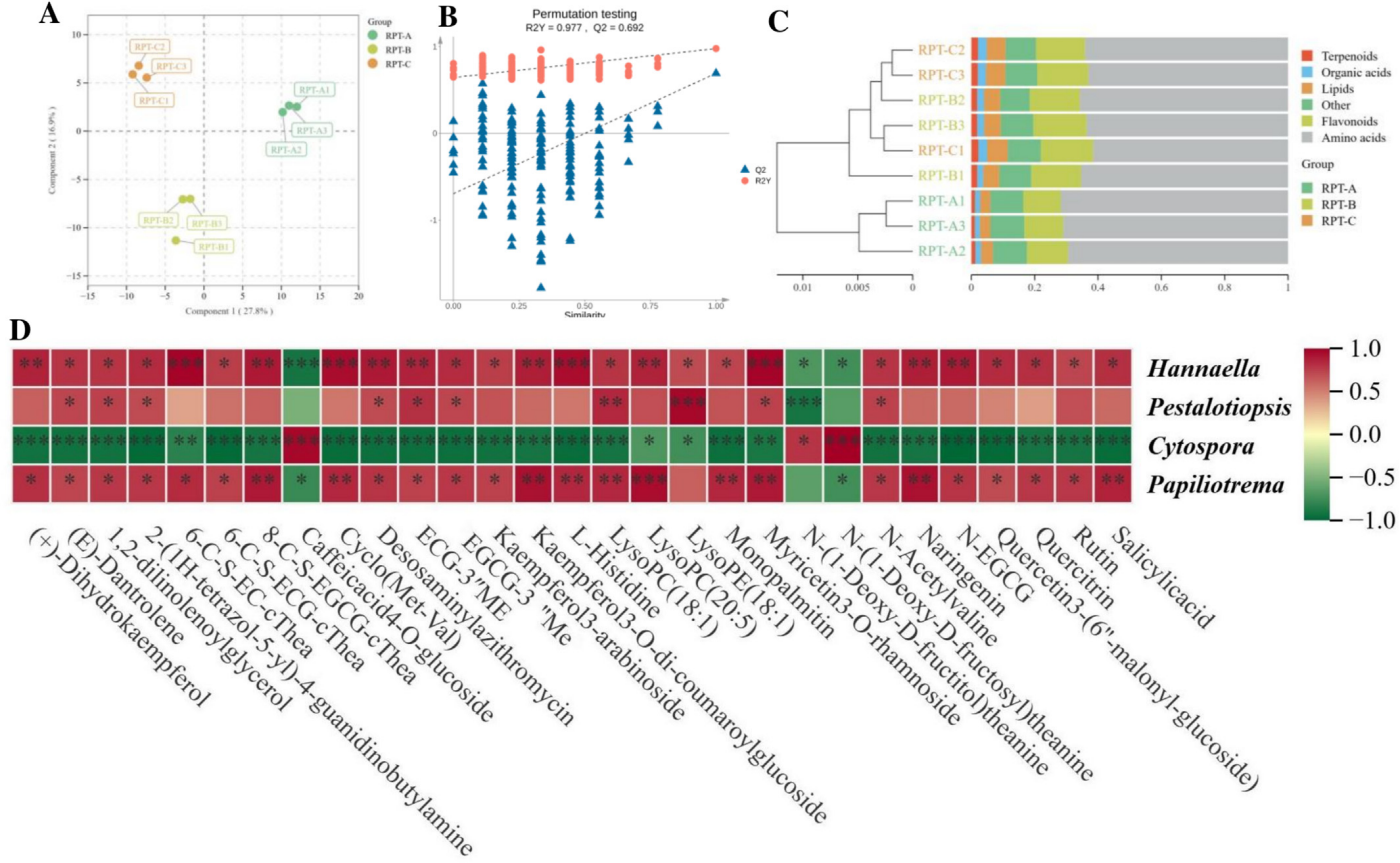
Changes in non-volatile metabolites (NVMs) of raw Pu-erh tea (RPT) stored under different water activity (A*w*) levels. Partial least squares discriminant analysis (PLS-DA) score plot and (B) Permutation test showing clear separation of three storage A*w* based on NVMs. (C) Hierarchical clustering of metabolite subclasses showing higher similarity between RPT-B and RPT-C than RPT-A. (D) Spearman correlations between fungal genera and NVMs (|*ρ*| > 0.90).

To investigate potential microbial influences on aroma differentiation, Spearman correlation analysis was conducted between fungal genera and A*w*-associated VMs (|*ρ*| > 0.80, Fig. 3F). According to Fig. 3F, *Papiliotrema* and *Hannaella* (both yeasts), along with *Toxicocladosporium*, showed strong positive correlations (*ρ* > 0.80, *p* < 0.05 or *p* < 0.01) with the accumulation of several aroma-active terpenoids in high-A*w* RPT samples, which mainly included monoterpenes such as terpinen-4-ol, *α*-terpineol, 3-caranol, and bornylene, the oxygenated monoterpene (*E*)-linalool oxide (furanoid), and sesquiterpenes such as *α*-bisabolol, *α*-calacorene, and cedrol. Similar associations between fungal genera and lipid oxidation-derived volatiles during natural aging of RPT have also been reported in vintage-based analyses [19]. These VMs originate from the isoprenoid (mevalonate) pathway, and their enrichment at higher A*w* suggests that microorganisms with heightened activity possibly *basidiomycetous* yeasts may channel acetyl-CoA through the mevalonate route to generate IPP and DMAPP, the universal precursors of mono- and sesquiterpenes, thereby contributing to terpenoid biosynthesis or release [44,51,52]. Conversely, *α*-ionone declined as A*w* increased and was negatively correlated with *Papiliotrema*, *Hannaella*, *Pestalotiopsis*, *Didymella*, and *Setophoma*. Previous studies have shown that certain fungi can hydroxylate or ketonize *α*-ionone through cytochrome P450- or peroxygenase-mediated oxidations, thereby depleting the parent compound under humid conditions [53]. In contrast, *β*-ionone showed no significant change across A*w* treatments, consistent with its lower susceptibility to such fungal transformations (Table S3). *Papiliotrema* and *Hannaella* showed positive correlations (*ρ* > 0.80, *p* < 0.05 or *p* < 0.01) with several non-terpenoid volatiles, including 2-acetoxy-5-hydroxyacetophenone, bourgeonal and 3-methyl-undecane, which can add sweet and floral notes to higher A*w* RPT [54]. However, together with *Acremonium*, they also correlated with several uncommon compounds, including phthalic acid butyl ester, *trans*-1,2-diphenylcyclobutane, and *N*,*N*-dimethyl-propanamide, which have been tentatively associated with plastic-like, fishy, or amine-like odors, and may pose potential sensory or safety concerns, although their exact impacts in tea matrices remain to be clarified [[55], [56], [57]]. Under humid storage, fungi can produce both pleasant and off-odor volatiles, and even within established microbial safety thresholds, excessive accumulation of these metabolites can still impair sensory quality.

Conversely, *Cytospora* was positively correlated (*ρ* > 0.80, *p* < 0.01 or *p* < 0.001) with several floral and green-note volatiles like 2-heptenoic acid 4-nitrophenyl ester, (*E*)-4-heptenal, benzaldehyde, (*E*,*E*)-2,4-heptadienal, isophthalaldehyde, 2-propyl-furan, methyl 2-methylpentanoate, and *α*-ionone, indicating a potential suppressive role (Fig. 3F). This further supports the notion that microbial succession contributes to lipid oxidation-related volatiles in aged RPT [19]. These findings highlighted the critical role of storage microenvironment, especially A*w*-mediated microbial activity, in shaping the characteristic aging aroma profile of RPT tea.

***Effects of Aw on microbial community shifts and NVM Profiles***.

***NVMs composition of stored RPT with different Aw***.

A total of 858 NVMs were detected in the stored RPT samples using UHPLC-Q-Exactive/MS in positive ESI mode. The PLS-DA score plot clearly separated RPT samples stored under different A*w* conditions (RPT-A, RPT-B, and RPT-C) (Fig. 3A). The model’s fit and predictive performance were further validated by permutation testing (R^2^Y = 0.977, Q^2^ = 0.692, *p* < 0.005) (Fig. 3B). Based on VIP > 1.0 and FDR-adjusted *q* < 0.05, 122 differential metabolites were identified and structurally annotated, including a wide range of flavonoids, amino acids, organic acids, lipids, and terpenoids compounds (Table S4). Notably, flavonoids, lipids, and amino acids were markedly affected by A*w*. As A*w* increased from 0.53 to 0.71, the relative abundance of flavonoids and lipids significantly increased (*q* < 0.05), whereas free amino acids showed a consistent decline. Specifically, flavonoids increased from 33 % in RPT-A to 44 % in RPT-C, while amino acids decreased from 56 % to 43 %.

In parallel, a wide range of flavonoids also declined, including flavonol glycosides like myricetin-3-*O*-glucoside, quercetin-3-*O*-glucoside, and kaempferol-3-*O*-rutinoside, as well as flavanol derivatives such as EGCG-3″Me, ECG-3″Me, *N*-EGCG, EC-(4*α* → 8)-ECG, Puerin B, and EGC-3,5-digallate. These increases suggest that methylated, conjugated, and galloylated flavonoids underwent microbial transformation under humid storage conditions (Fig. S2). Among umami-related compounds, several amino acids including *L*-arginine and *L*-theanine, along with theanine derivatives such as theanine glucoside, *N*-(1-deoxy-*D*-fructosyl)theanine, and *N*-(1-deoxy-*D*-fructitol)theanine, were significantly (*q* < 0.01) lower in RPT-C compared to RPT-A (Fig. S2). This metabolic reprogramming reflects typical aging-associated transformations in tea, where monomeric catechins and amino acids progressively degrade, while polymerized polyphenolic compounds accumulate over time [58]. The depletion of amino acids and flavonoids coincided with the formation of several flavoalkaloids, including 8-C-*S*-EC-cThea, 6-C-*S*-EC-cThea, and 8-C-*R*-EGCG-cThea, which are likely generated through condensation between amino acid degradation products and flavan-3-ols. Meanwhile, the abundance of peptide-type metabolites and *N*-acyl amino acids, such as cyclo(Met-Val), phenylalanine-threonine (Phe-Thr), and *N*-acetylvaline, progressively increased with rising A*w*. Together, these metabolic changes contributed to a sensory shift resembling RPT aging, which was characterized by reduced umami, bitterness, and astringency, along with enhanced mouthfeel and kokumi attributes [5,59]. Kokumi, described as a sensation of “thickness, mouthfulness, and continuity,” enhances the perception of basic tastes through kokumi-active compounds such as *γ*-glutamyl peptides, glutathione, and certain flavoalkaloids, which stimulate the calcium-sensing receptor (CaSR) in taste cells [[60], [61], [62]]. Widely identified in fermented foods, these compounds are increasingly recognized as key contributors to flavor enhancement in tea and as important indicators of quality improvement in aged RPT [5,63,64]. Concurrently, the accumulation of phospholipid degradation products such as LysoPC (18:1), LysoPE (18:2), and LPA (18:1) along with increased levels of phenolic acids and terpenoids, reflects enhanced microbial and oxidative metabolism under higher A*w* conditions [65].

Hierarchical clustering of metabolite subclasses (Fig. 3C) revealed greater similarity between RPT-B and RPT-C than with RPT-A, suggesting that moderate to high A*w* conditions resulted in more convergent metabolic profiles. These findings support the view that elevated humidity facilitates microbial activity and enzymatic transformation, which in turn promotes the accumulation of secondary metabolites such as flavonoids, phenolic acids, terpenoids, and lipids. This accumulation was likely driven by microbial activity at the expense of nitrogenous precursors, including free amino acids, reflecting a shift from primary nitrogen metabolism to polyphenol-derived secondary metabolism [66]. Overall, these results highlight storage A*w* as a key determinant of chemical evolution in RPT, with moderate to high A*w* levels favoring microbial-driven metabolic pathways associated with aging.

***Impact of fungal communities on NVMs during RPT Storage***.

Spearman correlation analysis was conducted to explore associations between fungal communities and NVMs in RPT under varying storage A*w* conditions. As shown in Fig. 3D, numerous strong correlations (|*ρ*| > 0.90) were observed between fungal genera and key metabolites, highlighting potential microbial contributions to chemical transformations during humid storage.

Notably, a cluster of flavoalkaloids such as 8-C-*S*-EGCG-cThea, 6-C-*S*-ECG-cThea and 6-C-*S*-EC-cThea exhibited significant positive correlations (*ρ* > 0.90, *p* < 0.05 or *p* < 0.01) with *Papiliotrema* and *Hannaella*, suggesting that these fungi may facilitate the condensation of flavan-3-ols with amino acid derivatives under elevated A*w*, thereby enhancing the kokumi attributes of aged RPT [5]. Such flavoalkaloids have previously been identified as marker compounds in dark teas and stored white teas [67]. Their formation is thought to proceed via Strecker degradation of *L*-theanine, followed by spontaneous cyclization of the intermediate and subsequent conjugation with EGCG, a mechanism verified to occur through both abiotic chemical reactions and fungal biosynthesis [68]. In parallel, multiple flavonol glycosides, including kaempferol 3-arabinoside, quercitrin, rutin, and myricetin 3-*O*-rhamnoside, were positively associated with *Papiliotrema*, and *Hannaella* (*ρ* > 0.90, *p* < 0.05), suggesting that these filamentous fungi may possess the activity of glycosidases or contribute to stabilization of glycosylated flavonoids [69]. These metabolites are known to modulate bitterness and astringency, potentially shaping the flavor profile of RPT during storage. In contrast, several amino acid derivatives, such as *N*-acetylvaline, *L*-histidine, and cyclo(Met-Val), exhibited significant positive correlations (*ρ* > 0.90, *p* < 0.05) with *Papiliotrema* and *Hannaella,* while caffeic acid 4-*O*-glucoside, *N*-(1-deoxy-*D*-fructosyl)theanine, and *N*-(1-deoxy-*D*-fructitol)theanine negative correlations (*ρ* < –0.9, *p* < 0.05) with the same taxa, indicating potential microbial consumption or transformation of nitrogenous precursors under high A*w* conditions. This may reflect fungal involvement in nitrogen remodeling during postfermentation [61]. Additionally, a group of lysophospholipids, including LysoPC (20:5), LysoPC (18:1), and LysoPE (18:1) were positively correlated (*ρ* > 0.90, *p* < 0.05, *p* < 0.01 or *p* < 0.001) with *Papiliotrema*, *Hannaella*, and *Pestalotiopsis*, which are known to possess phospholipase and lipid-degrading activities. This suggests that phospholipid breakdown is microbially mediated in moist storage environments, contributing to lipid remodeling [65].

Together, these findings demonstrate that fungal communities participate in the restructuring of the chemical profile of RPT during storage, with specific genera contributing to the formation or depletion of flavor-active metabolites, and possibly improving the microstructural organization of tea infusion particles that modulate mouthfeel [70].

***Verification of metabolic activity of RPT-derived fungi under simulated and realistic storage Aw conditions***.

***Isolation and identification of core fungi from stored RPT***.

**Fig. 4:**
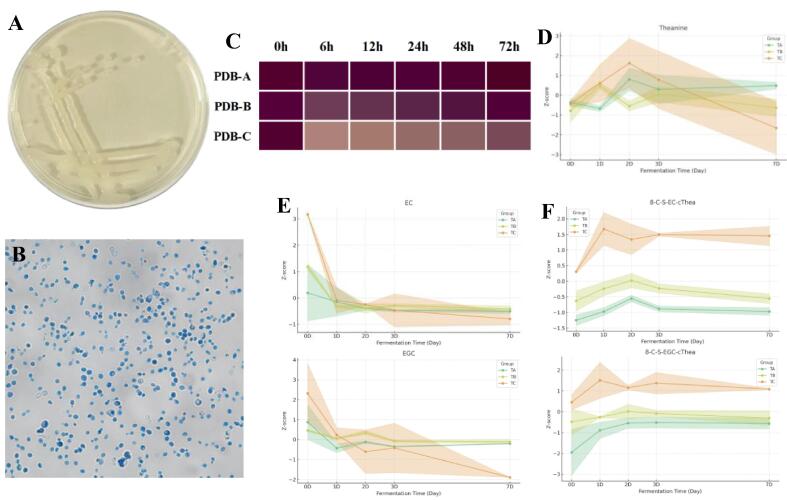
Identification and metabolic activity of *Papiliotrema flavescens* RPT (*P. flavescens* RPT) under simulated storage water activity (A*w*) conditions. Morphology of *P. flavescens* RPT colonies on PDA medium. (B) Microscopic morphology under cotton blue staining showing yeast-like budding. (C) Resazurin-based color difference (ΔE) indicating metabolic activity across A*w* levels. (D) Dynamic changes of theanine content in tea infusion during fermentation under different A*w* conditions (TA, TB, TC). (E) (−)-Epicatechin (EC) and (−)-epigallocatechin (EGC) showing significant decreases in TB and TC groups (*p* < 0.05). (F) Accumulation of flavoalkaloids 8-C-*S*-EC-cThea and 8-C-*S*-EGC-cThea over time in TB and TC groups.

The same yeast strain was consistently isolated from one-year stored RPT samples under all three A*w* conditions (0.53, 0.61, and 0.71). After 72 h of incubation on PDA medium at 28 °C, colony morphology analysis revealed pale yellow, mucoid colonies with glossy surfaces and irregular edges (Fig. 4A). Microscopic observation after cotton blue staining revealed round to oval cells with clear budding morphology, confirming the purity of the isolate and typical yeast-like features (Fig. 4B). Genomic DNA was extracted from purified colonies, and the ITS1-5.8S-ITS2 region was amplified using ITS1 and ITS4 primers. Agarose gel electrophoresis confirmed the presence of the target band (Fig. S2). Sequencing and BLAST analysis showed 99 % similarity to *Papiliotrema flavescens* (*P. flavescens*, GenBank Accession No. OQ415385.1), leading to the identification of the strain as *P. flavescens* RPT (GenBank Accession No. PV094664), belongs to the phylum *Basidiomycota*, class *Tremellomycetes*, order *Tremellales*, family *Rhynchogastremataceae*, and genus *Papiliotrema*. Growth characterization demonstrated that *P. flavescens* RPT was capable of growing on PDA plates supplemented with 4.5 M glycerol, 2.5 M sucrose, or 1 M sodium chloride, indicating strong osmotic tolerance and potential involvement in the transformation of flavor-active compounds during RPT storage.

As shown in Fig. 4C, *Papiliotrema* is a core functional yeast genus involved in flavor metabolics transformation during RPT storage. [4,5]. However, its association with off-odor compounds like *N*,*N*-dimethyl-propanamide suggests a need to balance its activity to avoid undesirable sensory outcomes.

***Metabolic activity of P. flavescens RPT under simulated storage conditions***.

RZ is an effective indicator of microbial metabolic activity, as it reflects microbial metabolism through the color change from blue RZ to pink resorufin (RF) and ultimately to colorless dihydroresorufin (hRF) upon reduction [38]. In this study, the metabolic activity of *P. flavescens* RPT under three different A*w* conditions was assessed by measuring the color change (ΔE) at various time points during fermentation.

The ΔE values were calculated and analyzed to evaluate the impact of A*w* on microbial metabolism (Table S5). The yeast cultured in PDB-A demonstrated negligible color change with the lowest ΔE values (5.22 ± 2.47) throughout fermentation, indicating its minimal metabolic activity and limited biochemical transformation (Fig. 4C). In contrast, PDB-B (A*w* 0.61) exhibited ΔE values approximately two- to threefold higher than those of PDB-A within the first 48 h, with the difference approaching significance (*p* = 0.0506). The most pronounced metabolic activity was observed in PDB-C (A*w* 0.71), which maintained the highest ΔE values (50.15 ± 9.89) across all conditions, and was significantly greater than both PDB-A and PDB-B (*p* < 0.001).

The observed positive correlation between A*w* and color change (ΔE) (Fig. 4C) demonstrates that *P. flavescens* RPT exhibits enhanced metabolic activity at elevated A*w* levels, which contributes to the formation of bioactive compounds such as kokumi-active metabolites under higher A*w* conditions. In contrast, PDB-A showed minimal metabolic activity, suggesting that A*w* below 0.6 significantly limits microbial metabolism, thereby slowing down the formation of flavor components of RPT.

***Metabolic impact of P. flavescens RPT on tea infusion under simulated storage conditions***.

Using *Z*-score normalization and time-resolved regression analysis (Pearson correlation and *p*-values), several representative tea-derived metabolites were identified whose dynamic changes were likely influenced by microbial activity. *L*-Theanine exhibited a complex temporal pattern during the first two days of fermentation, followed by a gradual decrease in both the TB and TC groups (*r* < – 0.2, *p* > 0.05, Fig. 4D). Although the time-dependent changes were not statistically significant, the final *L*-theanine content in the TC group was significantly lower than that in TA on day 7 (*p* = 0.032), suggesting delayed or limited microbial utilization of *L*-theanine, potentially due to preferential assimilation of alternative nitrogen sources. In contrast, epicatechin (EC), (−)-epigallocatechin (EGC) showed significant time-dependent decreases in the TB and TC groups (*r* < – 0.7, *p* < 0.05), with more pronounced declines observed under higher A*w* conditions (Fig. 4E). *L*-Theanine and flavan-3-ols such as EC and EGC are recognized as precursors for the formation of *N*-ethyl-2-pyrrolidinone-substituted flavanols (flavoalkaloids), which can be synthesized via fungal-mediated pathways during post-fermentation [71]. Two representative flavoalkaloids (i.e., 8-C-*S*-EC-cThea and 8-C-*S*-EGC-cThea) were detected, and exhibited slow accumulation during fermentation, particularly in the TB and TC groups (*r* > 0.2, *p* > 0.05, Fig. 4F). Although the accumulation trends over time were not statistically significant, their levels after three days were significantly higher than those in TA (*p* = 0.041). This may reflect the relatively low enzymatic activity of P. flavescens RPT under restricted A*w* conditions, while the group-dependent differences still supported the occurrence of microbial biotransformation at A*w* levels above 0.60.

Overall, these findings demonstrated that *P. flavescens* RPT participated in both the degradation and transformation of tea-derived compounds, potentially contributing to the flavor development of Pu-erh tea during post-fermentation under controlled A*w* conditions.

***Regulation of tea metabolites by P. flavescens RPT enzymes in realistic storage condition***

**Fig. 5:**
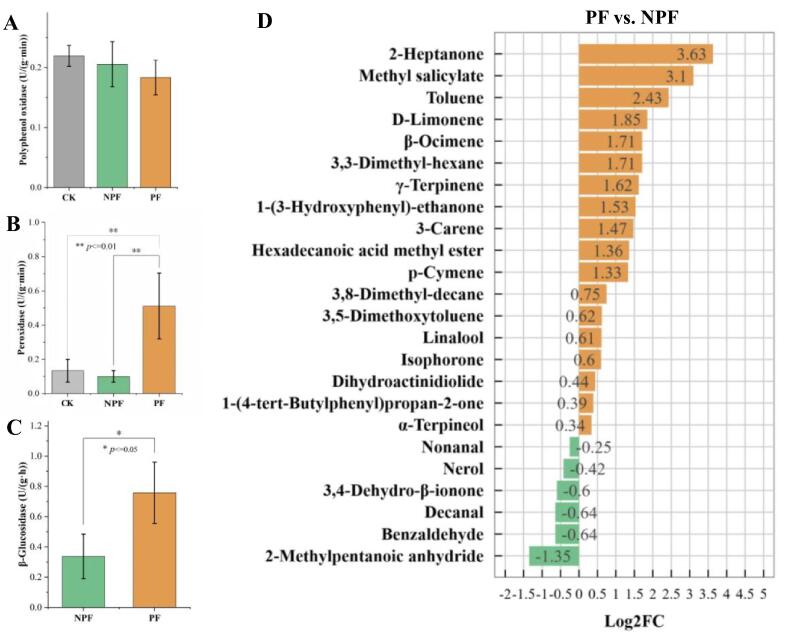
Enzyme production by *Papiliotrema flavescens* RPT (*P. flavescens* RPT) under realistic storage condition and its regulatory effects on tea metabolites. (A) Polyphenol oxidase (PPO) activity in *P. flavescens* RPT compared with the non-inoculated (NPF). (B) Peroxidase (POD) activity in *P. flavescens* RPT compared with NPF. (C) *β*-Glucosidase (*β*-GC) activity in *P. flavescens* RPT compared with NPF. (D) Differential volatile metabolites between *P. flavescens* RPT and NPF presented as log_2_ fold changes; positive values indicate up-regulation and negative values indicate down-regulation in the inoculated group.

To investigate the impact of microbial metabolic activity on tea flavor metabolites under realistic storage conditions, sterilized tea samples were inoculated with *P. flavescens* RPT (PF) and non-inoculated controls (NPF), and stored at 25 °C with an A*w* of 0.70–0.71 for 10 months. Enzymatic assays were performed on storage samples. As shown in Fig. 5A, PF exhibited no significant PPO activity relative to NPF or the boiled-inactivated enzyme control (CK), likely due to the absence of PPO in this yeast strain or its limited expression under storage conditions. In contrast, PF showed significantly higher POD activity (*p* = 0.008, Fig. 5B). Previous studies have suggested that POD catalyzes the hydroxylation of catechins and theaflavins to form tea pigments, thereby influencing the composition of flavor metabolites in tea [72]. Additionally, PF displayed significantly higher *β*-GC activity than NP (*p* = 0.044, Fig. 5C), and this enzyme hydrolyzes glycosidic bonds to release free aglycones that can subsequently undergo oxidation by POD or PPO, thereby contributing to the generation of flavor-active metabolites [73]. As illustrated in Fig. 5D, microbial metabolic activity in PF resulted in significant differences in 24 VMs (*p* < 0.05, FDR-adjusted *q* < 0.05). Aromatic compounds showed the strongest increase (log_2_FC = 2.62, ∼6-fold), followed by terpenoids (log_2_FC = 0.54, ∼1.5-fold), whereas aliphatics exhibited only a minor increase (log_2_FC = 0.27, ∼1.2-fold). Linalool was the most abundant VM in PF, reaching 2583.64 ± 191.64 μg/kg and increasing by approximately 893.57 μg/kg relative to NPF, while methyl salicylate reached 1213.33 ± 106.8 μg/kg, representing an 8.6-fold increase compared with NPF. These results strongly suggest that *P. flavescens* RPT, through *β*-GC accumulation, promoted the hydrolysis of glycosidic precursors and the release of linalool and methyl salicylate, consistent with previous findings that exogenous *β*-GC enhances aromatic compound formation [74].

In summary, under realistic storage conditions, microbial activity drives enzyme accumulation and subsequent metabolite formation. However, the detection of toluene and isophorone in PF, although at trace concentrations (18–21 μg/kg), suggested that when A*w* exceeded 0.70 such compounds might gradually accumulate, potentially leading to the development of low-grade characteristic or fungal-related volatiles during the long-term storage of RPT over decades [75,76].

## Conclusions

This study systematically evaluated the impact of A*w* on the microbial ecology and flavor chemistry of RPT during storage. Based on national moisture standards of RPT storage and Peleg model fitting, the safety threshold for A*w* was determined to be below 0.741.

Beyond merely ensuring safety, A*w* had a pronounced impact on both microbial community composition and metabolic activity during RPT storage. Higher A*w* levels promoted increased fungal abundance and diversity, with *Papiliotrema*, *Hannaella* and *Toxicocladosporium* were strongly associated with the accumulation of aging-associated volatiles, terpenoids including terpinen-4-ol, *α*-terpineol, *α*-bisabolol, *α*-calacorene, cedrol, linalool and its oxides. These results suggest that microbial activity under higher A*w* conditions accelerates the formation of aged aroma characteristics in RPT. In parallel, fungi such as *Papiliotrema*, *Hannaella*, *Didymella*, and *Pestalotiopsis* were closely linked to the accumulation of kokumi-active NVMs, including flavoalkaloids (i.e., 8-C-*S*-EC-cThea, 8-C-*R*-EGCG-cThea, and 6-C-*S*-EC-cThea) and amino acid derivatives (i.e., cyclo(Met-Val), Phe-Thr, and *N*-acetylvaline), which further enhanced the mouthfeel complexity.

A core yeast isolate, *P. flavescens* RPT, was identified from stored RPT and exhibited metabolic activity under both simulated and realistic storage conditions. Resazurin assays and the accumulation of flavoalkaloids demonstrated that microbial activity increased with rising A*w* above 0.60, providing direct evidence that indigenous microbes remain metabolically active under low-A*w* storage. Enzymatic assays of realistic storage samples (A*w* 0.70–0.71) further confirmed that *P. flavescens* produced *β*-GC, which hydrolyzed glycosidic precursors and led to the accumulation of aroma-active volatiles such as linalool and methyl salicylate, thus confirming a direct microbial contribution to flavor development. Collectively, these findings demonstrate that A*w* values above 0.60 progressively enhance microbial activity, leading to greater enzymatic functions and the formation of key flavor metabolites.

However, A*w* levels above 0.70 also promoted the formation of off-odor volatiles such as *N*,*N*-dimethyl-propanamide, toluene, and isophorone, indicating potential risks to sensory quality even within microbiological safety thresholds. Overall, this study establishes A*w* as a dual-purpose parameter that ensures storage safety while actively modulating microbial-driven flavor development, and identifies an optimal range of 0.60–0.70 that enables quality-controlled aging of RPT through targeted A*w* regulation. The framework established here provides the first theoretical basis for extending A*w* regulation to other tea types and for guiding large-scale storage practices to achieve controlled post-fermentation quality.

## Compliance with ethics requirements

This work did not involve any studies with human participants or animals. All experimental protocols, including microbial isolation and storage simulation, were conducted in accordance with institutional and international research ethics guidelines.

## Funding

This research was supported by Innovation Project for Chinese Academy of Agricultural Sciences (CAAS-ASTIP-TRI), Key Research and Development Program of Zhejiang Province (2025C01093).

## Declaration of competing interest

The authors declare that they have no known competing financial interests or personal relationships that could have appeared to influence the work reported in this paper.
