# Supplementary material for: New insights into optimal storage conditions for raw Pu-erh tea: modulating post-fermentation flavor via microbial metabolism under controlled water activity
Source: J Adv Res. 2025 Nov 5;86:15–27. doi: 10.1016/j.jare.2025.10.078 (PMC13453879; doi:10.1016/j.jare.2025.10.078)
Supplement: Supplementary file 1 [file mmc1.doc]

**Supplementary methods**

***Fungal ITS Sequencing and bioinformatic Analysis***

The ITS1 region was amplified using primers ITS5-1737F and ITS2-2043R with unique sample-specific barcodes. PCR amplification was performed in 15 μL reaction volumes containing 10 ng of template DNA, 0.2 μM of each primer, and Phusion® High-Fidelity PCR Master Mix (New England Biolabs, USA). The thermal cycling conditions were as follows: initial denaturation at 98 °C for 1 min; 30 cycles of 98 °C for 10 s, 50 °C for 30 s, and 72 °C for 30 s; and a final extension at 72 °C for 5 min. Following PCR product purification, quantification, and normalization, paired-end sequencing (2 × 250 bp) was conducted on the Illumina NovaSeq 6000 platform (Illumina Inc., San Diego, CA, USA). Each biological sample was analyzed in triplicate.

Raw reads were processed using FLASH for read merging, followed by quality control and denoising using DADA2 within QIIME2 to generate amplicon sequence variants (ASVs). Representative ASV sequences were taxonomically annotated using the UNITE database v8.2. ASV tables were rarefied to an equal sequencing depth of 6,046 reads per sample prior to *α*-diversity and *β*-diversity analyses. Bray–Curtis dissimilarity and principal coordinate analysis (PCoA) were used to visualize differences in fungal community structure across samples.

***GC–MS conditions and data processing***

GC–MS analysis was performed using an Agilent 6890 gas chromatograph coupled with an Agilent 5975 mass selective detector (Agilent Technologies, Wilmington, DE, USA), equipped with a DB-5MS capillary column (30 m × 0.25 mm × 0.25 μm, Agilent). High-purity helium (99.999%) was used as the carrier gas at a constant flow rate of 1.0 mL/min. The injector was maintained at 250 °C in splitless mode. The oven temperature program was as follows: held at 40 °C for 2 min, increased to 60 °C at 2 °C/min, then to 160 °C at 5 °C/min, and finally to 250 °C at 10 °C/min, held for 2 min. The mass spectrometer was operated in electron ionization (EI) mode at 70 eV, with the ion source temperature set at 230 °C. Mass spectra were acquired in full scan mode over an m/z range of 40–400. Compounds were identified against the NIST 2017 library with a similarity index ≥ 80%, features with > 50% missing values per group were excluded, and relative concentrations were calculated using the internal standard method.

***LC–MS conditions and data processing***

LC-MS analysis was conducted using a UHPLC-Q-Exactive/MS system (Thermo Fisher Scientific, Waltham, MA, USA) equipped with an Acquity UPLC HSS T3 column (2.1 mm × 100 mm, 1.8 μm, Waters, UK). The mobile phases consisted of 0.1% formic acid (v/v) and 0.1% trifluoroacetic acid (v/v) in water (A) and 0.1% formic acid in acetonitrile (B). The flow rate was 0.4 mL/min with the following gradient: 0–0.5 min, 2% B; 0.5–10.0 min, 2–15% B; 10.0–18.0 min, 15–40% B; 18.0–20.0 min, 40–90% B; 20.0–20.9 min, 90% B; and 21.0–25.0 min, 90–2% B. The column temperature was maintained at 40 °C and the autosampler at 4 °C.

Mass spectrometry was performed in positive electrospray ionization (ESI+) mode. The capillary temperature was set to 300 °C, the spray voltage to 3.5 kV, and the sheath and auxiliary gas flow rates to 40 and 10 arbitrary units, respectively. The drying gas temperature was 350 °C with a flow rate of 10 L/min. Full-scan data were acquired over an m/z range of 80–1200. To ensure data quality, features with a relative standard deviation (RSD) > 30% across QC samples were excluded, and the remaining peak areas were normalized to the total ion current (TIC) for comparability across samples.

***Strain identification details***

Genomic DNA was extracted from the purified isolates using the TSINGKE Plant DNA Extraction Kit (Tsingke Biotechnology, Beijing) following the manufacturer’s protocol. Briefly, samples were lysed with Buffer gP1 at 65 °C for 30 min, followed by precipitation with Buffer gP2. DNA was purified using a silica spin column, washed with ethanol-based buffers, and eluted in 50 μL of TE buffer (pH 8.0).

The internal transcribed spacer (ITS) region of fungal DNA was amplified using ITS1 (5′-TCCGTAGGTGAACCTGCGG-3′) and ITS4 (5′-TCCTCCGCTTATTGATATGC-3′) primers in a 50 μL PCR system containing TSE102 Gold Mix (Tsingke). The amplification protocol consisted of an initial denaturation at 98 °C for 2 min, followed by 35 cycles of 98 °C for 10 s, 54 °C for 10 s, and 72 °C for 10 s, with a final extension at 72 °C for 5 min. PCR products were visualized on agarose gel and purified for sequencing. The resulting sequences were aligned and taxonomically classified using the UNITE v9.0 database.

***Determination of enzyme activity***

A mixture of 0.5 g of tea powder, 0.9 g of polyvinylpyrrolidone (PVP), 0.03 g of quartz sand, and 5 mL of pre-cooled extraction buffer was homogenized in a mortar. The homogenate was incubated at 4 °C for 24 h and then centrifuged at 12,000 rpm for 10 min. The resulting supernatant, containing the crude enzyme extract, was collected for enzyme activity assays.

The activities of peroxidase (POD) and polyphenol oxidase (PPO) were measured using commercial assay kits (Shanghai Yuanye Bio-Technology Co., Ltd., Shanghai, China) following the manufacturer’s protocols. One unit of POD activity was defined as an increase in absorbance of 0.01 per minute due to guaiacol oxidation under the specified assay conditions. PPO activity was determined by monitoring the absorbance increase at 420 nm, with one unit defined as a 0.01 increase in absorbance per minute.

The activity of *β*-glucosidase (*β*-GC) was measured using an assay kit from Shanghai Acmec Biochemical Technology Co., Ltd. (Shanghai, China). One unit of *β*-GC activity was defined as the amount of enzyme required to produce 1 μmol of p-nitrophenol per hour under standard assay conditions at 37 °C.

**Table S1. Sorption isotherm models fitted to raw Pu-erh tea (RPT) samples at 20 °C.**

| Model | Fitted Equation | SSE | RMSE | R² | Adj. R² |
| --- | --- | --- | --- | --- | --- |
| Peleg | M_e_ = 10.06·A*_w_*^0.382^ + 45.70·A*_w_*^8.09^ | 12.71 | 0.495 | 0.9936 | 0.9931 |
| GAB | M_e_ = (3.954·2.786·0.959·A*_w_*) / ((1 - 0.959·A*_w_*)(1 - 0.959·A*_w_* + 2.786·0.959·A*_w_*)) | 33.26 | 0.800 | 0.9831 | 0.9824 |
| BET | M_e_ = (3.269·4.84·A*_w_*) / ((1 - A*_w_*)(1 - A*_w_* + 4.84·A*_w_*)) | 117.99 | 1.507 | 0.9401 | 0.9389 |
| Oswin | M_e_ = 7.770·(A*_w_* / (1 - A*_w_*))^0.577^ | 92.57 | 1.340 | 0.9530 | 0.9526 |
| Henderson | M_e_ = [−ln(1 − A*_w_*)]^{1/0.841} / 0.1025^ | 476.42 | 0.690 | 0.9894 | 0.9894 |
| Smith | M_e_ = 1.1788 − 10.1682·ln(1 − A*_w_*) | 182.03 | 1.877 | 0.9076 | 0.9057 |
| Halsey | Mₑ = (−6.5 / ln(A*_w_*))^(1 / 1.15)^ | 265.69 | 1.142 | 0.8569 | 0.8540 |

M_e_ is the equilibrium moisture content (%, dry basis); A*_w_* is the water activity. SSE is the sum of squared errors; RMSE is the root mean square error; R² is the coefficient of determination; Adj. R² is the adjusted R² value. Values represent the best-fit parameters derived from nonlinear regression.

Table S2. Effects of water activity (A*w*) on fungal α-diversity indices of raw Pu-erh tea (RPT) samples during storage.

| Genus | RPT-A (Mean±SD) | RPT-B (Mean±SD) | RPT-C (Mean±SD) |
| --- | --- | --- | --- |
| *Cladosporium* | 7633.3 ± 3133.2 a | 9684.7 ± 2117.7 a | 10368.7 ± 929.5 a |
| *Setophoma* | 2814.3 ± 1517.9 a | 3809.0 ± 1281.8 a | 5364.7 ± 2065.7 a |
| *Periconia* | 836.7 ± 556.9 a | 1091.0 ± 945.2 a | 946.3 ± 350.3 a |
| *Acrodontium* | 673.3 ± 258.9 a | 890.7 ± 474.6 a | 1208.0 ± 782.4 a |
| *Hannaella (yeast)* | 368.7 ± 327.2 a | 565.3 ± 340.6 a | 1272.3 ± 331.2 b |
| *Aureobasidium* | 418.3 ± 284.0 a | 688.0 ± 298.3 a | 894.3 ± 617.4 a |
| *Acremonium* | 10.3 ± 6.1 a | 1070.3 ± 1677.7 a | 633.7 ± 1031.2 a |
| *Toxicocladosporium* | 4.7 ± 8.1 a | 552.3 ± 952.3 a | 1093.7 ± 1690.6 a |
| *Pestalotiopsis* | 94.0 ± 113.2 a | 577.0 ± 434.5 a | 639.3 ± 575.0 a |
| *Didymella* | 175.3 ± 152.6 a | 427.7 ± 164.3 a | 539.0 ± 71.7 b |
| *Cytospora* | 624.7 ± 120.0 a | 295.0 ± 19.5 b | 142.0 ± 46.1 c |
| *Fusarium* | 276.3 ± 446.6 a | 112.3 ± 19.1 a | 652.0 ± 440.4 a |
| *Xenocamarosporium* | 467.7 ± 206.5 a | 190.3 ± 50.3 a | 275.7 ± 61.2 a |
| *Papiliotrema (yeast)* | 168.7 ± 138.5 a | 247.7 ± 84.89 a | 583.33 ± 221.81 b |
| *Wallemia* | 141.0 ± 168.5 a | 113.7 ± 141.6 a | 533.0 ± 648.7 a |
| *Phallus* | 122.0 ± 104.6 a | 538.7 ± 428.8 a | 95.3 ± 96.7 a |
| *Vishniacozyma (yeast)* | 111.0 ± 106.8 a | 436.3 ± 180.8 a | 180.7 ± 212.4 a |
| *Fungi_gen_Incertae_sedis* | 435.0 ± 224.6 a | 57.0 ± 53.0 a | 198.7 ± 269.1 a |
| *Capnodiales_gen_Incertae_sedis* | 62.3 ± 59.3 a | 242.3 ± 144.2 a | 358.7 ± 270.6 a |
| *Strelitziana* | 137.0 ± 146.2 a | 176.7 ± 82.6 a | 337.7 ± 311.2 a |

Values are expressed as mean ± standard deviation (SD, n = 3). Different lowercase letters (a, b, c) within the same row indicate significant differences among groups at *p* < 0.05 based on Tukey’s HSD test.

**Table S3.** Volatile metabolites (VMs) identified in raw Pu-erh tea (RPT) samples stored under different water activity (A*w*) conditions.

| **NO.** | **Classification** | **Formula** | **CAS NO.** | **Compounds** | **Relative Concentration (μg/kg)** | | | | |
| --- | --- | --- | --- | --- | --- | --- | --- | --- | --- |
|  |  |  |  |  | **RPT-A** | | **RPT-B** | | **RPT-C** |
| 1 | Aliphatic | C8H14O | 2548-87-0 | (E)-2-Octenal | 37.72±12.26 a | 25.45±5.23 a | | 16.02±1.52 b | |
| 2 | Aliphatic | C7H12O | 929-22-6 | (E)-4-Heptenal | 9.31±0.48 a | 4.24±0.58 B | | 3.04±0.67 B | |
| 3 | Aliphatic | C10H18O | 57283-79-1 | (E)-5-ethyl-6-methylhept-3-en-2-one | 18.49±3.73 a | 5.69±0.84 b | | 7.15±1.2 b | |
| 4 | Aliphatic | C7H10O | 4313-03-5 | (E,E)-2,4-Heptadienal | 78.39±5.19 a | 34.61±2.9 B | | 23.66±0.82 b | |
| 5 | Aliphatic | C8H12O | 30086-02-3 | (E,E)-3,5-Octadien-2-one | 23.66±4.32 a | 9.85±0.00* b | | 16.1±2.79 b | |
| 6 | Aliphatic | C4H6O3 | 16088-62-3 | (S)-Methyl-oxirane | 3.91±0.65 a | 4.72±0.13 a | | 4.59±0.03 a | |
| 7 | Aliphatic | C8H16O | 26001-58-1 | (Z)-2-Octen-1-ol | 30.56±8.06 | 37.01±6.0 | | nd | |
| 8 | Aliphatic | C7H12O | 2918-13-0 | 1-Hepten-3-one | 22.08±9.99 a | 22.21±6.09 a | | 15.47±2.27 a | |
| 9 | Aliphatic | C8H18O | 111-87-5 | 1-Octanol | 30.49±11.13 a | 26.77±3.25 a | | 21.35±3.54 a | |
| 10 | Aliphatic | C8H16O | 3391-86-4 | 1-Octen-3-ol | 125.35±50.41 a | 125.51±25.19 a | | 92.57±15.99 a | |
| 11 | Aliphatic | C5H8O | 1629-58-9 | 1-Penten-3-one | 10.36±0.7 a | 3.56±0.73 B | | 3.92±0.76 B | |
| 12 | Aliphatic | C16H30O4 | 6846-50-0 | 2,2,4-Trimethyl-1,3-pentanediol diisobutyrate | 50.59±34.65 a | 44.54±23.2 a | | 64.36±42.84 a | |
| 13 | Aliphatic | C9H20 | C9H20 | 2,4,4-Trimethyl-hexane | 1.89±0.34 a | 1.98±0.44 a | | 1.63±0.27 a | |
| 14 | Aliphatic | C16H34 | 3891-99-4 | 2,6,10-Trimethyltridecane | 7.88±0.39 a | 11.73±1.7 a | | 7.77±2.76 a | |
| 15 | Aliphatic | C15H32 | 504-44-9 | 2,6,11,15-Tetramethyl-hexadecane | 13.39±6.78 a | 37.43±16.85 a | | 20.75±0.61 a | |
| 16 | Aliphatic | C7H12 | 5750/2/7 | 2-Cyclopropyl-butane | 7.47±1.68 a | 5.23±0.56 a | | 3.8±0.86 b | |
| 17 | Aliphatic | C8H18O | 104-76-7 | 2-Ethyl-1-hexanol | 23.58±2.04 a | 24.15±1.75 a | | 16.31±2.09 b | |
| 18 | Aliphatic | C16H34O | 1000115-66-1 | 2-Ethyl-2-methyl-tridecanol | 9.76±6.29 | 5.06±1.38 | | nd | |
| 19 | Aliphatic | C12H24O2 | 1000369-74-6 | 2-Ethylbutyric acid 3-methylpent-2-yl ester | 4.94±0.58 a | 2.88±1.21 b | | 3.37±0.11 a | |
| 20 | Aliphatic | C7H14O | 110-43-0 | 2-Heptanone | 6.42±2.99 a | 5.91±0.12 a | | 8.76±0.68 a | |
| 21 | Aliphatic | C5H10O | 96-17-3 | 2-Methyl-butanal | 3.65±0.24 a | 4.2±0.34 a | | 2.77±0.48 b | |
| 22 | Aliphatic | C12H22O3 | 63169-61-9 | 2-methylpentanoic anhydride | 14.92±5.32 a | 17.79±1.44 a | | 14.46±5.87 a | |
| 23 | Aliphatic | C10H20O2 | 67801-33-6 | 3-(Hydroxymethyl)-2-nonanone | 5.33±0.59 a | 7.57±1.51 a | | 6.39±0.33 a | |
| 24 | Aliphatic | C8H18 | 563-16-6 | 3,3-Dimethyl-hexane | 7.37±2.78 a | 8.71±4.94 a | | 16.75±7.75 a | |
| 25 | Aliphatic | C9H18 | 26456-76-8 | 3,5,5-Trimethyl-2-hexene | 14.07±9.46 a | 17.46±3.27 a | | 11.15±3.34 a | |
| 26 | Aliphatic | C8H12O | 38284-27-4 | 3,5-Octadien-2-one | 116.46±43.74 a | 12.2±1.76 b | | 15.46±1.5 b | |
| 27 | Aliphatic | C12H26 | 17312-53-7 | 3,6-Dimethyl-decane | 6.71±0.94 a | 6.23±0.45 a | | 23.05±5.52 b | |
| 28 | Aliphatic | C16H31N | 17312-55-9 | 3,8-Dimethyl-decane | 7.79±6.91 a | 8.27±0.94 a | | 4.22±1.97 a | |
| 29 | Aliphatic | C12H26 | 17302-01-1 | 3-Ethyl-3-methylheptane | 14.63±6.56 a | 19.22±14.84 a | | 10.45±5.88 a | |
| 30 | Aliphatic | C11H22O3 | 77-68-9 | 3-Hydroxy-2,2,4-trimethylpentyl isobutyrate | 5.14±0.56 a | 2.12±0.22 B | | 4.2±0.36 b | |
| 31 | Aliphatic | C5H10O | 590-86-3 | 3-Methyl-butanal | 1.87±0.67 a | 2.53±1.17 a | | 2.21±0.54 a | |
| 32 | Aliphatic | C7H10N2O2 | 6418-44-6 | 3-Methyl-heptadecane | 2.2±0.23 a | 3.39±0.53 b | | 2.0±0.09 c | |
| 33 | Aliphatic | C5H6N4O2 | 2882-96-4 | 3-Methyl-pentadecane | nd | 6.17±2.0 | | 5.98±1.3 | |
| 34 | Aliphatic | C25H49NO4 | 6418-41-3 | 3-Methyl-tridecane | 3.12±0.59 a | 3.83±1.73 a | | 4.04±1.79 a | |
| 35 | Aliphatic | C16H26O | 1002-43-3 | 3-Methyl-undecane | nd | 5.29±0.1 | | 8.6±2.55 | |
| 36 | Aliphatic | C14H24O5 | 17302-23-7 | 4,5-Dimethyl-nonane | 31.11±5.05 a | 30.17±7.07 a | | 20.29±3.05 a | |
| 37 | Aliphatic | C6H15Al | 6008-17-9 | 5,5-Dibutylnonane | 6.78±2.37 a | 13.02±1.94 b | | 8.44±1.32 a | |
| 38 | Aliphatic | C10H20O | 112-31-2 | Decanal | 40.74±7.93 a | 34.13±10.28 a | | 38.46±5.76 a | |
| 39 | Aliphatic | C16H28 | 112-40-3 | Dodecane | 16.08±6.21 a | 7.43±2.83 a | | 13.54±2.9 a | |
| 40 | Aliphatic | C8H10O4 | 629-94-7 | Heneicosane | 55.04±11.55 a | 44.74±21.6 a | | 40.37±4.34 a | |
| 41 | Aliphatic | C7H14O | 111-71-7 | Heptanal | 30.88±10.88 a | 25.25±3.69 a | | 20.0±2.86 a | |
| 42 | Aliphatic | C5H7NO2 | 544-76-3 | Hexadecane | 13.3±0.25 a | 28.57±11.2 a | | 27.23±9.19 a | |
| 43 | Aliphatic | C18H36O2 | 628-97-7 | Hexadecanoic acid ethyl ester | 2.3±2.55 a | 2.52±2.03 a | | 6.18±0.97 a | |
| 44 | Aliphatic | C17H34O2 | 112-39-0 | Hexadecanoic acid methyl ester | 4.01±0.17 a | 3.45±0.94 a | | 10.33±2.19 b | |
| 45 | Aliphatic | C6H12O | 66-25-1 | Hexanal | 36.16±13.89 a | 29.3±4.09 a | | 20.12±4.01 a | |
| 46 | Aliphatic | C17H34O2 | 110-27-0 | Isopropyl myristate | 0.99±0.4 a | 1.01±0.32 a | | 1.14±0.23 a | |
| 47 | Aliphatic | C7H14O2 | 2177-77-7 | Methyl 2-methylpentanoate | 8.69±0.5 a | 7.58±0.6 a | | 6.52±0.65 b | |
| 48 | Aliphatic | C5H11NO | 758-96-3 | N,N-Dimethyl-propanamide | nd | nd | | 16.41±3.97 | |
| 49 | Aliphatic | C40H84N2 | 2883/2/5 | n-Nonylcyclohexane | 1.98±0.13 a | 4.1±1.3 b | | 2.19±0.54 a | |
| 50 | Aliphatic | C9H18O | 124-19-6 | Nonanal | 114.33±18.11 a | 102.01±9.37 a | | 105.26±3.11 a | |
| 51 | Aliphatic | C18H36O2 | 1000406-06-0 | Nonyl 3,5,5-trimethylhexanoate | 89.53±24.79 a | 45.49±19.26 a | | 37.65±11.42 b | |
| 52 | Aliphatic | C19H40 | 1921-70-6 | Norphytane | 2.44±0.00* a | 12.65±3.91 b | | 8.22±2.33 a | |
| 53 | Aliphatic | C6H9NO | 3892-00-0 | Norpristane | nd | 15.23±4.58 | | 9.33±2.74 | |
| 54 | Aliphatic | C18H38 | 593-45-3 | Octadecane | nd | 9.51±3.08 | | 20.44±14.56 | |
| 55 | Aliphatic | C8H16O | 124-13-0 | Octanal | 36.26±8.67 a | 38.34±6.84 a | | 31.07±1.71 a | |
| 56 | Aliphatic | C2H2O4 | 144-62-7 | Oxalic acid | 8.71±0.27 | 5.13±4.63 | | 5.55±0.84 | |
| 57 | Aliphatic | C15H32 | 629-62-9 | Pentadecane | 13.22±0.88 a | 20.78±9.16 a | | 16.12±5.25 a | |
| 58 | Aliphatic | C5H10O | 110-62-3 | Pentanal | 5.74±0.15 a | 6.77±1.29 a | | 4.69±1.16 a | |
| 59 | Aliphatic | C14H28O2 | 1000406-05-5 | Pentyl 3,5,5-trimethylhexanoate | 14.59±4.55 a | 11.72±0.6 a | | 9.29±0.76 a | |
| 60 | Aliphatic | C20H42 | 638-36-8 | Phytane | 7.22±1.43 a | 12.61±4.8 a | | 30.36±12.41 b | |
| 61 | Aliphatic | C14H30 | 629-59-4 | Tetradecane | 17.14±1.92 a | 16.61±6.03 a | | 12.64±2.0 a | |
| 62 | Aliphatic | C13H28 | 629-50-5 | Tridecane | 32.76±27.57 a | 23.04±2.76 a | | 22.42±2.82 a | |
| 63 | Aromatic | C9H12O | 70424-13-4 | (Z)-2-(2-Pentenyl)furan | 5.44±1.96 a | 3.17±0.31 a | | 2.45±0.37 b | |
| 64 | Aromatic | C13H16O2 | 25152-85-6 | (Z)-3-Hexenyl benzoate | 7.7±1.58 a | 5.7±0.6 a | | 7.96±0.57 a | |
| 65 | Aromatic | C9H12O | 536-50-5 | 1-(4-Methylphenyl)ethanol | 7.02±0.42 a | 6.69±0.57 a | | 6.97±0.43 a | |
| 66 | Aromatic | C13H16 | 53156-11-9 | 1,2-Dihydro-4,5,7-Trimethylnaphthalene | 1.73±0.3 a | 1.66±0.05 a | | 2.24±0.27 a | |
| 67 | Aromatic | C8H10 | 108-38-3 | 1,3-Dimethyl-benzene | 5.14±2.17 a | 4.37±1.15 a | | 6.53±2.66 a | |
| 68 | Aromatic | C16H18 | 3075-84-1 | 2,2',5,5'-TETRAMETHYLBIPHENYL | 5.35±0.13 a | 24.09±1.2 B | | 8.71±0.5 C | |
| 69 | Aromatic | C14H21NO3 | 20039-94-5 | 2,4-Bis(tert-butyl)-6-nitrophenol | 2.57±3.11 a | 4.98±4.05 a | | 2.95±2.54 a | |
| 70 | Aromatic | C19H32O3 | 166273-38-7 | 2,4-Ditert-butylphenyl 5-hydroxypentanoate | 19.64±4.03 a | 22.64±4.61 a | | 19.17±2.08 a | |
| 71 | Aromatic | C17H26O2 | 14035-34-8 | 2,6-Bis(1,1-dimethylethyl)-4-(1-oxopropyl)phenol | 1.48±0.43 a | 1.62±0.66 a | | 1.6±0.88 a | |
| 72 | Aromatic | C10H10O4 | 144152-29-4 | 2-Acetoxy-5-hydroxyacetophenone | 3.02±0.14 a | 3.52±0.28 a | | 4.89±0.81 b | |
| 73 | Aromatic | C7H8O2 | 1193-79-9 | 2-Acetyl-5-methylfuran | 5.06±0.08 a | 3.36±0.3 B | | 5.96±0.22 C | |
| 74 | Aromatic | C13H17NO1 | 1000406-90-6 | 2-Heptenoic acid 4-nitrophenyl ester | 5.87±2.45 | 2.35±0.31 | | nd | |
| 75 | Aromatic | C5H6O2 | 25414-22-6 | 2-Methoxy-furan | 8.96±4.04 a | 12.27±1.74 a | | 7.69±1.78 a | |
| 76 | Aromatic | C11H10 | 91-57-6 | 2-Methyl-naphthalene | 4.7±0.67 a | 5.03±0.27 a | | 4.24±0.12 a | |
| 77 | Aromatic | C7H10O | 4229-91-8 | 2-Propyl-furan | 35.19±6.75 a | 28.35±0.1 a | | 15.96±2.25 b | |
| 78 | Aromatic | C12H13NO1 | 1000307-59-8 | 3-Methylbut-2-enoic acid, 4-nitrophenyl ester | 4.67±0.38 a | 3.83±2.28 a | | 1.43±0.00* a | |
| 79 | Aromatic | C34H50O4 | 1000354-54-8 | 4-(2-Phenylpropyl-2)-phenyl sebacic acid nonyl ester | 0.8±0.17 a | 0.93±0.05 a | | 0.56±0.12 b | |
| 80 | Aromatic | C10H12 | 27831-13-6 | 4-Ethenyl-1,2-dimethyl-benzene | 3.97±2.07 a | 3.14±0.14 a | | 3.57±0.19 a | |
| 81 | Aromatic | C13H18O | 25552-17-4 | 5-Methyl-1-phenyl-1-hexanone | 4.31±0.71 a | 6.56±1.54 a | | 3.15±0.00* b | |
| 82 | Aromatic | C13H26O2 | 500-66-3 | 5-Pentyl-1,3-benzenediol | 128.92±20.51 a | 104.14±12.06 a | | 126.03±16.13 a | |
| 83 | Aromatic | C8H8O | 98-86-2 | Acetophenone | 5.92±3.31 a | 7.68±1.86 a | | 14.76±9.77 a | |
| 84 | Aromatic | C7H6O | 100-52-7 | Benzaldehyde | 167.37±35.86 a | 104.89±9.96 b | | 82.45±10.59 b | |
| 85 | Aromatic | C8H8O | 122-78-1 | Benzeneacetaldehyde | 69.12±24.74 a | 75.44±11.38 a | | 74.24±9.26 a | |
| 86 | Aromatic | C13H18O | 18127-01-0 | Bourgeonal | 5.22±0.23 a | 7.19±2.2 a | | 11.54±2.15 b | |
| 87 | Aromatic | C3H4N2O | 128-37-0 | Butylated Hydroxytoluene | 60.02±3.36 a | 48.09±1.01 b | | 81.12±1.64 B | |
| 88 | Aromatic | C8H10 | 100-41-4 | Ethylbenzene | 4.72±0.55 a | 4.88±0.62 a | | 8.48±2.49 a | |
| 89 | Aromatic | C8H6O2 | 626-19-7 | Isophthalaldehyde | 9.98±3.48 a | 5.26±1.08 a | | 3.05±0.3 b | |
| 90 | Aromatic | C8H9NO2 | 1000222-86-6 | Methoxy-phenyl-oxime | 43.32±3.39 a | 54.29±6.6 a | | 57.68±7.67 a | |
| 91 | Aromatic | C21H16N2O2 | 3510-24-5 | Methyl p-(2-phenyl-1-benzimidazolyl)benzoate | 1.87±0.31 a | 1.55±0.22 a | | 1.78±0.78 a | |
| 92 | Aromatic | C8H8O3 | 119-36-8 | Methyl salicylate | 80.49±19.88 a | 41.68±3.19 a | | 274.06±30.55 B | |
| 93 | Aromatic | C10H8 | 91-20-3 | Naphthalene | 12.31±1.09 a | 11.4±0.44 a | | 10.92±0.96 a | |
| 94 | Aromatic | C20H16O5 | 1000330-45-8 | O-(2-Furoyl)-O'-(3-phenylpropionyl)-1,2-benzenediol | 1.22±0.22 a | 1.28±0.28 a | | 1.49±0.72 a | |
| 95 | Aromatic | C16H22O4 | 1000315-17-4 | Phthalic acid butyl ester | nd | 5.19±0.75 | | 3.81±2.61 | |
| 96 | Aromatic | C20H30O4 | 1000314-84-7 | Phthalic acid isobutyl 4-octyl ester | 5.85±0.02 a | 7.35±1.48 a | | 10.48±2.28 b | |
| 97 | Aromatic | C8H8 | 100-42-5 | Styrene | 47.54±5.22 a | 15.58±6.23 b | | 59.02±11.51 c | |
| 98 | Aromatic | C7H9NO | 2167-14-8 | Tea pyrrole | 56.87±17.91 a | 55.5±7.06 a | | 44.9±7.24 a | |
| 99 | Aromatic | C7H8 | 108-88-3 | Toluene | 9.95±0.8 a | 9.99±0.76 a | | 6.98±0.86 b | |
| 100 | Aromatic | C4H5NO2 | 20071-09-4 | trans-1,2-Diphenylcyclobutane | nd | nd | | 12.85±1.03 | |
| 101 | Terpenoid | C10H18O2 | 34995-77-2 | (E)-Linalool oxide (furanoid) | nd | nd | | 65.0±8.9 | |
| 102 | Terpenoid | C10H18O2 | 39028-58-5 | (E)-Linalool oxide (pyranoid) | 3.06±0.32 a | 2.06±0.18 b | | 4.12±0.14 B | |
| 103 | Terpenoid | C10H18O2 | 5989-33-3 | (Z)-Linalool Oxide | 15.42±9.24 a | 9.32±0.84 a | | 53.76±1.88 B | |
| 104 | Terpenoid | C24H39NO4 | 1000143-97-2 | 2-(Dimethylamino)ethyl (2E)-(3-hydroxy-14-methyl-7-oxopodocarpan-13-ylidene)ethanoate | 7.2±0.06 a | 6.24±0.9 a | | 4.69±2.67 a | |
| 105 | Terpenoid | C9H16O | 2408-37-9 | 2,2,6-Trimethyl-cyclohexanone | 44.36±10.31 a | 55.67±6.65 a | | 51.66±9.9 a | |
| 106 | Terpenoid | C9H14O | 17299-41-1 | 3,4,4-Trimethyl-2-cyclohexen-1-one | 4.32±1.6 a | 4.19±0.65 a | | 5.5±0.66 a | |
| 107 | Terpenoid | C10H18O | 4017-79-2 | 3-Caranol | 19.19±1.03 a | 20.96±0.73 a | | 23.71±2.02 b | |
| 108 | Terpenoid | C12H18O2 | 23267-57-4 | 4-(2,2,6-Trimethyl-7-oxabicyclo[4.1.0]hept-1-yl)-3-buten-2-one | 35.48±6.12 a | 27.87±2.56 a | | 39.1±6.78 a | |
| 109 | Terpenoid | C13H20O | 17283-81-7 | 4-(2,6,6-Trimethyl-1-cyclohexen-1-yl)-2-butanone | 2.33±0.26 a | 1.47±0.11 b | | 2.46±0.4 c | |
| 110 | Terpenoid | C18H36O | 502-69-2 | 6,10,14-Trimethyl-2-pentadecanone | 11.97±1.65 a | 13.25±4.05 a | | 17.98±2.46 a | |
| 111 | Terpenoid | C8H14O | 110-93-0 | 6-Methyl-5-hepten-2-one | 43.93±3.6 a | 34.95±14.84 a | | 45.79±8.35 a | |
| 112 | Terpenoid | C10H14 | 464-17-5 | Bornylene | 9.37±1.29 a | 10.61±2.69 a | | 22.34±2.84 b | |
| 113 | Terpenoid | C15H26O | 77-53-2 | Cedrol | 26.73±5.58 a | 43.63±4.46 b | | 51.77±6.72 b | |
| 114 | Terpenoid | C11H16O2 | 17092-92-1 | Dihydroactinidiolide | 28.08±2.77 a | 19.35±1.77 b | | 17.27±1.83 b | |
| 115 | Terpenoid | C10H16 | 5989-27-5 | D-Limonene | 145.52±12.34 a | 162.55±3.83 a | | 165.18±5.27 a | |
| 116 | Terpenoid | C15H24 | 41702-63-0 | Epizonarene | 1.12±0.55 a | 1.87±0.55 a | | 2.26±0.00* b | |
| 117 | Terpenoid | C13H22O | 3796-70-1 | Geranyl acetone | 122.19±2.94 a | 89.16±10.95 b | | 109.63±4.88 c | |
| 118 | Terpenoid | C10H16O | 29957-43-5 | Hotrienol | 35.56±14.49 a | 53.63±9.36 a | | 36.39±8.75 a | |
| 119 | Terpenoid | C9H14O | 78-59-1 | Isophorone | 19.25±5.2 a | 17.93±4.53 a | | 17.25±2.78 a | |
| 120 | Terpenoid | C10H18O | 78-70-6 | Linalool | 455.37±104.92 a | 337.71±44.75 a | | 878.15±118.34 b | |
| 121 | Terpenoid | C20H38 | 504-96-1 | Neophytadiene | 7.6±0.07 a | 8.19±0.6 a | | 7.45±0.19 a | |
| 122 | Terpenoid | C10H14 | 527-84-4 | o-Cymene | 34.79±0.00* a | 44.25±0.49 B | | 40.6±2.16 b | |
| 123 | Terpenoid | C10H14O | 116-26-7 | Safranal | 61.82±12.41 a | 61.53±6.89 a | | 61.35±7.18 a | |
| 124 | Terpenoid | C10H18O | 562-74-3 | Terpinen-4-ol | 11.67±2.53 a | 19.27±1.52 b | | 26.73±3.07 c | |
| 125 | Terpenoid | C13H22O | 36431-72-8 | Theaspirane | 12.65±1.15 a | 19.45±1.72 b | | 20.61±1.68 b | |
| 126 | Terpenoid | C15H26O | 23178-88-3 | α-Bisabolol | nd | 10.67±1.36 | | 15.35±1.41 | |
| 127 | Terpenoid | C15H20 | 21391-99-1 | α-Calacorene | 1.02±0.02 a | 1.29±0.13 b | | 1.87±0.09 B | |
| 128 | Terpenoid | C13H20O | 127-41-3 | α-Ionone | 176.22±25.24 a | 132.87±11.0 b | | 118.46±0.00* b | |
| 129 | Terpenoid | C10H18O | 98-55-5 | α-Terpineol | 78.91±20.83 a | 105.1±10.24 a | | 154.39±17.42 b | |
| 130 | Terpenoid | C10H16O | 432-25-7 | β-Cyclocitral | 186.83±24.99 a | 154.8±16.52 a | | 155.68±17.24 a | |
| 131 | Terpenoid | C11H18O | 472-66-2 | β-Homocyclocitral | 8.33±1.84 a | 6.7±1.16 a | | 28.18±1.88 B | |
| 132 | Terpenoid | C13H20O | 14901-07-6 | β-Ionone | 434.19±65.61 a | 308.01±9.64 b | | 350.56±33.83 a | |
| 133 | Terpenoid | C10H16 | 123-35-3 | β-Myrcene | nd | nd | | 52.11±4.66 | |
| 134 | Terpenoid | C10H16 | 99-85-4 | γ-Terpinene | 9.98±2.08 a | 12.5±3.17 a | | 14.46±1.56 a | |
|  | **Aliphatics** |  |  |  | 1309.5 ± 216.3 a | 1114.7 ± 167.7 a | | 1006.6 ± 125.1 a | |
|  | **Aromatics** |  |  |  | 843.3 ± 132.1 ab | 705.6 ± 52.5 b | | 991.6 ± 127.3 a | |
|  | **Terpenoids** |  |  |  | 2054.5 ± 318.1 ab | 1797.1 ± 147.9 b | | 2661.8 ± 266.4 a | |
|  | **Total** |  |  |  | 4207.3 ± 662.2 a | 3617.4 ± 345.6 a | | 4660.1 ± 506.6 a | |

Values are expressed as relative concentrations calculated by the internal standard method and presented as mean ± standard deviation (SD, n = 3). Different lowercase letters (a, b, c) indicate significant differences between groups at p < 0.05, while uppercase letters (A, B, C) indicate highly significant differences at p < 0.01, based on Tukey’s HSD test. “nd” denotes a value of zero, and “*” indicates an approximately zero standard deviation. All displayed p-values were adjusted using the FDR correction.

**Table S4.** Differential Non-volatile metabolites (NVMs) identified in raw Pu-erh tea (RPT) stored under different water activity (A*w*) conditions.

| **No.** | **Classification** | **Name** | **Formula** | **Exact mass** | **RT [min]** | **Relative abundance** | | |
| --- | --- | --- | --- | --- | --- | --- | --- | --- |
|  |  |  |  |  |  | **RPT-A** | **RPT-B** | **RPT-C** |
| 1 | Amino acids | (2S,3S)‑2,3‑Dihydro‑3‑hydroxy‑anthranilic acid | C7H9NO3 | 155.05857 | 1.267 | 0.000106 ± 0.000025a | 0.000137 ± 0.000027A | 0.000268 ± 0.000036A |
| 2 | Amino acids | L-Histidine | C6H9N3O2 | 155.0586 | 3.517 | 0.000070 ± 0.000004a | 0.000083 ± 0.000009A | 0.000139 ± 0.000009A |
| 3 | Amino acids | N-Acetylvaline | C7H13NO3 | 159.09 | 5.114 | 0.000139 ± 0.000004a | 0.000186 ± 0.000004A | 0.000261 ± 0.000012A |
| 4 | Amino acids | L-Theanine | C7H14N2O3 | 174.10082 | 1.287 | 0.040888 ± 0.002486A | 0.030784 ± 0.001691a | 0.031393 ± 0.002205A |
| 5 | Amino acids | L-Anginine | C7H14N2O3 | 174.10095 | 1.097 | 0.032690 ± 0.001730a | 0.027379 ± 0.002636a | 0.024541 ± 0.001246b |
| 6 | Amino acids | N-Acetyl-S-methylcysteine | C7H15NO2S | 177.08267 | 1.637 | 0.000378 ± 0.000006A | 0.000268 ± 0.000026A | 0.000098 ± 0.000006a |
| 7 | Amino acids | Leucyl‑Proline | C11H20N2O3 | 228.13419 | 14.558 | 0.000379 ± 0.000039a | 0.001460 ± 0.000160A | 0.000942 ± 0.000110A |
| 8 | Amino acids | Cyclo(Met-Val) | C10H18N2O2S | 230.12685 | 1.538 | 0.000211 ± 0.000011A | 0.000244 ± 0.000018a | 0.000271 ± 0.000006A |
| 9 | Amino acids | 2-(1H-tetrazol-5-yl)-4-guanidinobutylamine | C12H17NO3 | 239.15011 | 15.525 | 0.000369 ± 0.000012a | 0.000477 ± 0.000011A | 0.000705 ± 0.000027A |
| 10 | Amino acids | (E)-Cinnamoyl-agmatine | C11H20N2O5 | 260.16406 | 13.729 | 0.000422 ± 0.000020a | 0.000454 ± 0.000032A | 0.000262 ± 0.000022A |
| 11 | Amino acids | Phenylalanine-Threonine | C13H18N2O4 | 266.12702 | 6.981 | 0.000351 ± 0.000056A | 0.000571 ± 0.000061a | 0.000704 ± 0.000095A |
| 12 | Amino acids | N-Phenylacetylphenylalanine | C17H17NO3 | 283.10353 | 6.126 | 0.000223 ± 0.000016A | 0.000162 ± 0.000017A | 0.000161 ± 0.000015a |
| 13 | Amino acids | Indole-3-acetyl-L-leucine | C16H20N2O3 | 288.11892 | 3.414 | 0.000459 ± 0.000007A | 0.000573 ± 0.000010A | 0.000569 ± 0.000019a |
| 14 | Amino acids | Theanineglucoside | C13H24N2O8 | 336.15363 | 1.207 | 0.001012 ± 0.000154A | 0.000545 ± 0.000026A | 0.000468 ± 0.000040a |
| 15 | Amino acids | N-(1-Deoxy-D-fructosyl)theanine | C14H20N6O4 | 336.15363 | 0.798 | 0.000102 ± 0.000004A | 0.000054 ± 0.000011A | 0.000037 ± 0.000006a |
| 16 | Amino acids | N-(1-Deoxy-D-fructitol)theanine | C15H23N5O2S | 337.15712 | 1.206 | 0.000587 ± 0.000034A | 0.000253 ± 0.000065A | 0.000205 ± 0.000050a |
| 17 | Flavonoids | Naringenin | C15H12O5 | 272.06886 | 14.257 | 0.000056 ± 0.000005a | 0.000168 ± 0.000006A | 0.000240 ± 0.000012A |
| 18 | Flavonoids | Epiafzelechin | C15H14O5 | 274.08449 | 11.523 | 0.001087 ± 0.000019b | 0.001564 ± 0.000083a | 0.001830 ± 0.000154a |
| 19 | Flavonoids | Phloretin | C15H14O5 | 274.1032 | 1.763 | 0.000775 ± 0.000050A | 0.000995 ± 0.000010A | 0.000932 ± 0.000080a |
| 20 | Flavonoids | Kaempferol | C15H10O6 | 286.04807 | 14.245 | 0.000964 ± 0.000047A | 0.001210 ± 0.000046A | 0.001121 ± 0.000047a |
| 21 | Flavonoids | (+)-Dihydrokaempferol | C15H12O6 | 288.06366 | 12.254 | 0.000059 ± 0.000005a | 0.000337 ± 0.000010A | 0.000498 ± 0.000026A |
| 22 | Flavonoids | (–)-Dihydrokaempferol | C15H12O6 | 288.06368 | 13.25 | 0.000366 ± 0.000037A | 0.000348 ± 0.000016a | 0.000257 ± 0.000013A |
| 23 | Flavonoids | 5,7-Dimethoxy-4'-hydroxyflavanone | C16H14O6 | 300.11228 | 7.359 | 0.000751 ± 0.000048A | 0.000709 ± 0.000117a | 0.000337 ± 0.000047A |
| 24 | Flavonoids | EGC | C15H14O7 | 306.10833 | 10.842 | 0.000445 ± 0.000040A | 0.000618 ± 0.000070a | 0.000652 ± 0.000044A |
| 25 | Flavonoids | GC | C15H14O7 | 306.14282 | 1.289 | 0.000093 ± 0.000003A | 0.000050 ± 0.000005A | 0.000025 ± 0.000003a |
| 26 | Flavonoids | Myricetin | C15H10O8 | 318.03788 | 12.004 | 0.000590 ± 0.000023a | 0.000572 ± 0.000015b | 0.000708 ± 0.000057a |
| 27 | Flavonoids | 4'-O-Methylkievitone | C21H22O6 | 370.16069 | 9.272 | 0.000197 ± 0.000008A | 0.000180 ± 0.000024a | 0.000052 ± 0.000005A |
| 28 | Flavonoids | PuerinA | C22H24O7 | 400.15029 | 12.38 | 0.000052 ± 0.000002A | 0.000065 ± 0.000005a | 0.000071 ± 0.000004A |
| 29 | Flavonoids | 8-C-S-EC-cThea | C21H23NO7 | 401.14804 | 12.899 | 0.000178 ± 0.000033a | 0.000212 ± 0.000010A | 0.000388 ± 0.000009A |
| 30 | Flavonoids | 6-C-S-EC-cThea | C21H23NO7 | 401.14823 | 12.245 | 0.000092 ± 0.000011a | 0.000103 ± 0.000010A | 0.000175 ± 0.000023A |
| 31 | Flavonoids | PuerinB | C21H12N4O6 | 416.07504 | 12.224 | 0.000200 ± 0.000007a | 0.000203 ± 0.000005A | 0.000161 ± 0.000004A |
| 32 | Flavonoids | 8-C-R-EGC-cThea | C21H23NO8 | 417.143 | 11.403 | 0.000156 ± 0.000051b | 0.000172 ± 0.000043a | 0.000332 ± 0.000040a |
| 33 | Flavonoids | Kaempferol3-arabinoside | C20H18O10 | 418.24904 | 21.148 | 0.000031 ± 0.000007b | 0.000042 ± 0.000005a | 0.000067 ± 0.000009a |
| 34 | Flavonoids | [Epiafzelechin3-O-gallate](https://pubchem.ncbi.nlm.nih.gov/compound/467295) | C22H18O9 | 426.09561 | 14.244 | 0.002001 ± 0.000298A | 0.003247 ± 0.000086A | 0.003038 ± 0.000033a |
| 35 | Flavonoids | Isovitexin | C21H20O10 | 432.10639 | 13.194 | 0.000260 ± 0.000012a | 0.000274 ± 0.000007A | 0.000199 ± 0.000002A |
| 36 | Flavonoids | Vitexin | C21H20O10 | 432.10642 | 12.993 | 0.000270 ± 0.000007A | 0.000266 ± 0.000018a | 0.000205 ± 0.000009A |
| 37 | Flavonoids | 3-(4-Hydroxy­benzoyl)-epicatechin | C21H18O11 | 438.11657 | 9.254 | 0.000288 ± 0.000015A | 0.000287 ± 0.000016a | 0.000388 ± 0.000010A |
| 38 | Flavonoids | Galloyl-catechinA | C20H22O11 | 440.07503 | 13.258 | 0.000376 ± 0.000021a | 0.000396 ± 0.000006A | 0.000285 ± 0.000010A |
| 39 | Flavonoids | Olmesartan | [C24H26N6O3](https://pubchem.ncbi.nlm.nih.gov/#query=C24H26N6O3) | 446.20595 | 9.356 | 0.001626 ± 0.000279b | 0.002100 ± 0.000280a | 0.003033 ± 0.000346a |
| 40 | Flavonoids | Quercitrin | C21H20O11 | 448.10123 | 13.028 | 0.000020 ± 0.000001A | 0.000024 ± 0.000002a | 0.000027 ± 0.000002A |
| 41 | Flavonoids | ECG-3″ME | C17H21N4O9P | 456.10635 | 14.255 | 0.000098 ± 0.000008a | 0.000776 ± 0.000009A | 0.001143 ± 0.000054A |
| 42 | Flavonoids | N-EGCG | C12H19NO10 | 457.10163 | 7.214 | 0.000831 ± 0.000047b | 0.001292 ± 0.000045a | 0.001515 ± 0.000085a |
| 43 | Flavonoids | Myricetin3-O-rhamnoside | C21H20O12 | 464.05975 | 8.385 | 0.001773 ± 0.000143A | 0.002071 ± 0.000078a | 0.002233 ± 0.000082A |
| 44 | Flavonoids | Quercetin3-O-galactoside | C21H20O12 | 464.09627 | 13.151 | 0.001759 ± 0.000112A | 0.002166 ± 0.000042A | 0.001985 ± 0.000044a |
| 45 | Flavonoids | Quercetin3-O-glucosidea | C21H20O12 | 464.09628 | 13.349 | 0.003214 ± 0.000130A | 0.003922 ± 0.000117A | 0.003781 ± 0.000041a |
| 46 | Flavonoids | Quercetin4'-O-glucoside | C21H20O12 | 464.25471 | 21.464 | 0.000129 ± 0.000027A | 0.000203 ± 0.000030a | 0.000242 ± 0.000030A |
| 47 | Flavonoids | EGCG-3"Me | C23H20O11 | 472.10144 | 12.259 | 0.000166 ± 0.000006a | 0.001040 ± 0.000018A | 0.001584 ± 0.000043A |
| 48 | Flavonoids | Myricetin3-O-glucoside | C21H20O13 | 480.09131 | 12.005 | 0.001095 ± 0.000076a | 0.001109 ± 0.000028A | 0.001329 ± 0.000015A |
| 49 | Flavonoids | Dihydromyricetin3-O-glucoside | C21H22O13 | 482.26576 | 21.456 | 0.000122 ± 0.000017A | 0.000193 ± 0.000018a | 0.000220 ± 0.000017A |
| 50 | Flavonoids | Quercetin3-(6''-malonyl-glucoside) | C25H34N2O9 | 506.06822 | 7.873 | 0.000627 ± 0.000025A | 0.000741 ± 0.000012a | 0.000800 ± 0.000053A |
| 51 | Flavonoids | 6″-O-Acetylisoquercitrin | C25H34N2O9 | 506.10704 | 11.997 | 0.000263 ± 0.000014A | 0.000346 ± 0.000014a | 0.000361 ± 0.000002A |
| 52 | Flavonoids | Apigenindi-C-arabinoside | C25H26O13 | 534.13842 | 13.253 | 0.000917 ± 0.000003A | 0.001342 ± 0.000110A | 0.001181 ± 0.000036a |
| 53 | Flavonoids | Apigenindi-C-xyloside | C25H26O13 | 534.13844 | 13.823 | 0.000218 ± 0.000010a | 0.000369 ± 0.000014A | 0.000293 ± 0.000005A |
| 54 | Flavonoids | 6-C-S-ECG-cThea | C28H27NO11 | 553.1595 | 14.157 | 0.000160 ± 0.000003a | 0.000190 ± 0.000004A | 0.000453 ± 0.000018A |
| 55 | Flavonoids | 8-C-R-ECG-cThea | C28H27NO11 | 553.15956 | 14.554 | 0.000103 ± 0.000008b | 0.000135 ± 0.000023a | 0.000367 ± 0.000124a |
| 56 | Flavonoids | 8-C-R-EGCG-cThea | C28H27NO12 | 569.15447 | 13.164 | 0.000347 ± 0.000038a | 0.000431 ± 0.000011A | 0.000800 ± 0.000008A |
| 57 | Flavonoids | 8-C-S-EGCG-cThea | C28H27NO12 | 569.15449 | 12.602 | 0.000207 ± 0.000010a | 0.000253 ± 0.000010A | 0.000459 ± 0.000011A |
| 58 | Flavonoids | ProcyanidinB2 | C30H26O12 | 578.27729 | 20.711 | 0.000262 ± 0.000025A | 0.000409 ± 0.000049a | 0.000455 ± 0.000055A |
| 59 | Flavonoids | [Kaempferol3-O-rutinoside](https://pubchem.ncbi.nlm.nih.gov/compound/5318767) | C27H30O15 | 594.15958 | 12.698 | 0.000372 ± 0.000004A | 0.000368 ± 0.000009a | 0.000306 ± 0.000016A |
| 60 | Flavonoids | Kempferol3-O-glucosyl(1-2)rhamnoside | C27H30O15 | 594.2823 | 20.716 | 0.000095 ± 0.000014a | 0.000158 ± 0.000010A | 0.000239 ± 0.000033A |
| 61 | Flavonoids | [Naringenin5,7-di-O-glucoside](https://pubchem.ncbi.nlm.nih.gov/compound/42607912) | C27H32O15 | 596.29743 | 20.982 | 0.000086 ± 0.000004a | 0.000160 ± 0.000011A | 0.000236 ± 0.000009A |
| 62 | Flavonoids | EGC-3,5-digallate | C29H22O15 | 610.0968 | 13.249 | 0.000464 ± 0.000012a | 0.000473 ± 0.000024A | 0.000363 ± 0.000022A |
| 63 | Flavonoids | Rutin | C27H30O16 | 610.15438 | 13.099 | 0.004204 ± 0.000134b | 0.004597 ± 0.000052a | 0.004954 ± 0.000127a |
| 64 | Flavonoids | Quercetin3-(2-galloylglucoside) | C28H24O16 | 616.1073 | 8.993 | 0.000233 ± 0.000006A | 0.000217 ± 0.000014a | 0.000151 ± 0.000010A |
| 65 | Flavonoids | 8-C-ascorbylepigallocatechin-3-gallate | C28H24O17 | 632.10234 | 6.956 | 0.000226 ± 0.000004a | 0.000236 ± 0.000014A | 0.000119 ± 0.000012A |
| 66 | Flavonoids | EC-(4α→8)-ECG | C38H26N4O12 | 730.15438 | 11.27 | 0.002009 ± 0.000046a | 0.002203 ± 0.000076a | 0.001719 ± 0.000041b |
| 67 | Flavonoids | Gallocateaflavin3‑O‑gallate | C36H29O16N | 731.15779 | 11.269 | 0.000952 ± 0.000043a | 0.001050 ± 0.000063A | 0.000818 ± 0.000025A |
| 68 | Flavonoids | Oolongtheanin | C36H28O17 | 732.16055 | 11.269 | 0.000312 ± 0.000004a | 0.000337 ± 0.000021A | 0.000257 ± 0.000003A |
| 69 | Flavonoids | Kaempferol3-O-di-coumaroylglucoside | C39H32O15 | 740.17536 | 19.541 | 0.000278 ± 0.000014a | 0.000299 ± 0.000008A | 0.000357 ± 0.000019A |
| 70 | Flavonoids | Kaempferol3-O-[pC-Rha-Rha-Gal] | C43H42N4O17 | 886.25473 | 16.336 | 0.000509 ± 0.000015A | 0.000423 ± 0.000013a | 0.000444 ± 0.000011A |
| 71 | Lipids | Palmiticacid | C16H32O2 | 256.07393 | 14.235 | 0.000408 ± 0.000022A | 0.000635 ± 0.000017A | 0.000625 ± 0.000038a |
| 72 | Lipids | [Monopalmitin](https://pubchem.ncbi.nlm.nih.gov/compound/14900) | C19H38O4 | 330.27742 | 21.283 | 0.000157 ± 0.000011b | 0.000197 ± 0.000023a | 0.000256 ± 0.000027a |
| 73 | Lipids | 1-α-linolenoyl-glycerol | C21H36O4 | 352.26171 | 21.215 | 0.001631 ± 0.000161b | 0.002398 ± 0.000103a | 0.002845 ± 0.000187a |
| 74 | Lipids | 2-α-linolenoyl-glycerol | C21H36O4 | 352.26189 | 20.851 | 0.000447 ± 0.000011a | 0.000834 ± 0.000014A | 0.001064 ± 0.000057A |
| 75 | Lipids | [CyclicPhosphatidicacid](https://pubchem.ncbi.nlm.nih.gov/compound/51040919) | C21H41O6P | 420.26479 | 21.159 | 0.000167 ± 0.000024A | 0.000017 ± 0.000003a | 0.000019 ± 0.000006A |
| 76 | Lipids | LPA(18:1) | C20H18O11 | 434.24403 | 21.67 | 0.000067 ± 0.000010a | 0.000101 ± 0.000005A | 0.000133 ± 0.000008A |
| 77 | Lipids | Cer(d20:2/10:0) | C21H44NO7P | 453.28628 | 21.346 | 0.000659 ± 0.000047A | 0.000854 ± 0.000074a | 0.000991 ± 0.000058A |
| 78 | Lipids | LysoPE(18:3) | C23H42NO7P | 475.27081 | 20.905 | 0.000304 ± 0.000043a | 0.000438 ± 0.000042A | 0.000625 ± 0.000034A |
| 79 | Lipids | LysoPI(16:0) | C25H49O12P | 476.2644 | 15.33 | 0.000224 ± 0.000019A | 0.000315 ± 0.000020a | 0.000346 ± 0.000019A |
| 80 | Lipids | LysoPE(18:2) | C23H44NO7P | 477.2866 | 21.148 | 0.000887 ± 0.000059b | 0.001296 ± 0.000079a | 0.001752 ± 0.000264a |
| 81 | Lipids | LysoPE(18:1) | C23H46NO7P | 479.30235 | 21.483 | 0.000149 ± 0.000024A | 0.000262 ± 0.000044a | 0.000297 ± 0.000010A |
| 82 | Lipids | LysoPE(20:5） | C25H42NO7P | 499.2686 | 21.145 | 0.000199 ± 0.000028b | 0.000282 ± 0.000026a | 0.000372 ± 0.000024a |
| 83 | Lipids | LysoPC(17:1) | C25H50NO7P | 508.28117 | 21.39 | 0.000075 ± 0.000014A | 0.000106 ± 0.000018a | 0.000135 ± 0.000011A |
| 84 | Lipids | LysoPC(18:3) | C26H48NO7P | 517.31804 | 20.951 | 0.000890 ± 0.000105a | 0.001292 ± 0.000188A | 0.001951 ± 0.000098A |
| 85 | Lipids | LPC(18:2/0:0) | C26H50NO7P | 519.26097 | 20.851 | 0.000092 ± 0.000014b | 0.000151 ± 0.000015a | 0.000194 ± 0.000018a |
| 86 | Lipids | PC(0:0/18:2(9Z,12Z)) | C26H50NO7P | 519.33369 | 21.223 | 0.002070 ± 0.000248b | 0.002825 ± 0.000644a | 0.004042 ± 0.000354a |
| 87 | Lipids | LysoPC(18:1) | C26H52NO7P | 521.34935 | 21.618 | 0.000361 ± 0.000040A | 0.000620 ± 0.000048a | 0.000737 ± 0.000055A |
| 88 | Lipids | LPC18:1 | C26H52NO7P | 521.34943 | 21.508 | 0.000234 ± 0.000016b | 0.000282 ± 0.000024a | 0.000361 ± 0.000036a |
| 89 | Lipids | LysoPC(18:0) | C21H51N9O2P2 | 523.36502 | 21.122 | 0.000060 ± 0.000009b | 0.000102 ± 0.000014a | 0.000144 ± 0.000018a |
| 90 | Lipids | LysoPC(20:5) | C28H48NO7P | 541.31536 | 21.207 | 0.000373 ± 0.000073b | 0.000471 ± 0.000100a | 0.000722 ± 0.000078a |
| 91 | Lipids | LPI(16:0) | C25H49O12P | 572.29753 | 21.173 | 0.000175 ± 0.000017b | 0.000216 ± 0.000016a | 0.000268 ± 0.000024a |
| 92 | Lipids | 1,2-dilinolenoylglycerol | C39H64O5 | 612.16011 | 13.1 | 0.000439 ± 0.000011b | 0.000471 ± 0.000019a | 0.000529 ± 0.000024a |
| 93 | Lipids | 1,3-dilinolenoylglycerol | C39H64O5 | 612.41943 | 20.924 | 0.000424 ± 0.000031A | 0.000167 ± 0.000019a | 0.000198 ± 0.000031A |
| 94 | Lipids | PC (18:3/20:4) | C46H84NO9P | 826.52135 | 21.391 | 0.000125 ± 0.000021A | 0.000050 ± 0.000017a | 0.000056 ± 0.000019A |
| 95 | Organic acids | Benzoic acid | C7H6O2 | 122.03721 | 14.262 | 0.000022 ± 0.000004a | 0.000183 ± 0.000005A | 0.000279 ± 0.000007A |
| 96 | Organic acids | Salicylic acid | C7H6O3 | 138.03204 | 11.635 | 0.000028 ± 0.000001b | 0.000033 ± 0.000002a | 0.000040 ± 0.000002a |
| 97 | Organic acids | 3-Hydroxytetradecanedioicacid | C14H26O5 | 274.17964 | 14.206 | 0.000320 ± 0.000042a | 0.000321 ± 0.000022A | 0.000203 ± 0.000010A |
| 98 | Organic acids | [5-O-caffeoylshikimate](https://pubchem.ncbi.nlm.nih.gov/compound/25243950) | C16H15O8 | 335.0669 | 2.49 | 0.000763 ± 0.000059a | 0.000842 ± 0.000062A | 0.001222 ± 0.000111A |
| 99 | Organic acids | Asterric acid | C17H16O8 | 348.08257 | 8.025 | 0.000715 ± 0.000029a | 0.000654 ± 0.000063b | 0.000867 ± 0.000039a |
| 100 | Organic acids | Chicoricacid | C22H18O12 | 474.0809 | 9.203 | 0.000132 ± 0.000011A | 0.000200 ± 0.000013a | 0.000216 ± 0.000002A |
| 101 | Organic acids | 5-O-Caffeoylquinicacidglucoside | C22H30O13 | 502.18498 | 15.918 | 0.000045 ± 0.000002a | 0.000032 ± 0.000001b | 0.000037 ± 0.000004a |
| 102 | Organic acids | 3-O-Caffeoylquinicacidglucoside | C22H30O13 | 502.18505 | 14.386 | 0.000393 ± 0.000018a | 0.000401 ± 0.000012A | 0.000332 ± 0.000018A |
| 103 | Other | 1H-Inden-1-one | C5H14N4 | 130.04221 | 7.429 | 0.003240 ± 0.000129a | 0.002682 ± 0.000114b | 0.002899 ± 0.000147a |
| 104 | Other | Cinnamylalcohol | C9H10O | 134.07355 | 19.637 | 0.000181 ± 0.000015b | 0.000194 ± 0.000015a | 0.000252 ± 0.000021a |
| 105 | Other | Coumarin | C9H6O2 | 146.03714 | 9.032 | 0.000872 ± 0.000043A | 0.000780 ± 0.000062a | 0.000578 ± 0.000026A |
| 106 | Other | Methylcinnamate | C10H10O2 | 162.0684 | 7.429 | 0.003973 ± 0.000189a | 0.003092 ± 0.000185b | 0.003488 ± 0.000157a |
| 107 | Other | Methylthioadenosine | C11H15N5O3S | 297.08984 | 6.784 | 0.002000 ± 0.000296A | 0.001612 ± 0.000310a | 0.000971 ± 0.000136A |
| 108 | Other | (E)-Dantrolene | C14H10N4O5 | 314.06419 | 7.874 | 0.000923 ± 0.000040b | 0.001023 ± 0.000099a | 0.001198 ± 0.000020a |
| 109 | Other | (2R)-2-(β-D-Glucopyranosyloxy)-2-phenylacetamide | C14H19NO7 | 335.09852 | 4.165 | 0.000060 ± 0.000002a | 0.000064 ± 0.000004A | 0.000113 ± 0.000005A |
| 110 | Other | [Caffeicacid4-O-glucoside](https://pubchem.ncbi.nlm.nih.gov/compound/156579987) | C15H18O9 | 342.12277 | 10.168 | 0.001588 ± 0.000111A | 0.001041 ± 0.000072A | 0.000552 ± 0.000048a |
| 111 | Other | 2′‑Deoxycoformycin‑5′‑phosphate | C11H17N4O7P | 348.08255 | 8.544 | 0.000513 ± 0.000041A | 0.000503 ± 0.000035a | 0.000335 ± 0.000025A |
| 112 | Other | Glycyrol | C21H18O6 | 366.11892 | 7.424 | 0.000452 ± 0.000007a | 0.000375 ± 0.000018b | 0.000396 ± 0.000025a |
| 113 | Other | StigmatellinY | C29H40O6 | 484.2812 | 21.677 | 0.000127 ± 0.000005A | 0.000204 ± 0.000013a | 0.000216 ± 0.000014A |
| 114 | Other | Desosaminylazithromycin | C30H58N2O9 | 590.12834 | 12.367 | 0.000190 ± 0.000005a | 0.000219 ± 0.000015A | 0.000265 ± 0.000014A |
| 115 | Other | AntiarosideB | C35H54O14 | 698.35069 | 20.856 | 0.000604 ± 0.000102b | 0.001136 ± 0.000148a | 0.001506 ± 0.000144a |
| 116 | Other | E-Astringin | C20H22O9 | 406.10939 | 1.775 | 0.000058 ± 0.000007a | 0.000092 ± 0.000007A | 0.000176 ± 0.000012A |
| 117 | Other | (1S)-1,5-anhydro-2-O-[(E)-2-(3,4-dihydroxyphenyl)ethen-1-yl]-1-(1,3,6,7-tetrahydroxy-9-oxo-9H-xanthen-2-yl)-D-glucitol | C27H24O13 | 556.12035 | 13.263 | 0.000273 ± 0.000014A | 0.000394 ± 0.000046A | 0.000369 ± 0.000019a |
| 118 | Other | 4,4',4''-(Ethane-1,1,1-triyl)tris[2,6-bis(ethoxymethyl)phenol] | C38H54O9 | 654.37549 | 20.896 | 0.000299 ± 0.000021a | 0.000234 ± 0.000015b | 0.000241 ± 0.000016a |
| 119 | Terpenoids | 3‑Hydroxy‑9,10‑dinor‑6‑megastigmen‑8,5‑olide | C7H8N4O3 | 196.11004 | 12.945 | 0.000108 ± 0.000011a | 0.000120 ± 0.000005A | 0.000083 ± 0.000011A |
| 120 | Terpenoids | (E,E)-Farnesylacetone | C10H20N4O4 | 260.21428 | 21.243 | 0.000299 ± 0.000039A | 0.000460 ± 0.000029a | 0.000525 ± 0.000011A |
| 121 | Terpenoids | Brusatol | C26H32O11 | 520.19544 | 10.238 | 0.000290 ± 0.000022a | 0.000220 ± 0.000013b | 0.000245 ± 0.000009a |
| 122 | Terpenoids | 4-Ketomyxol | C40H54O4 | 598.40315 | 21.667 | 0.000158 ± 0.000003a | 0.000176 ± 0.000006A | 0.000137 ± 0.000012A |

Values are expressed as relative abundances obtained from peak area normalization and presented as mean ± standard deviation (SD, n = 3). Only NVMs that satisfied both false discovery rate (FDR) correction (adjusted *p* < 0.05) and variable importance in projection (VIP > 1.0, OPLS-DA model) were retained for presentation. Different lowercase letters (a, b, c) indicate significant differences between groups at *p* < 0.05, while uppercase letters (A, B, C) indicate highly significant differences at *P* < 0.01, according to Tukey’s HSD test.

**Table S5.** Color difference (ΔE) values of the resazurin reduction assay during *P. flavescens* RPT fermentation under different water activity (A*w*) conditions.

|  | PDB-A | | | | PDB-B | | | | PDB-C | | | |
| --- | --- | --- | --- | --- | --- | --- | --- | --- | --- | --- | --- | --- |
| Time | L | a | b | ΔE | L | a | b | ΔE | L | a | b | ΔE |
| 0h | 14.21 | 43.57 | -6.67 | 0.00 | 16.57 | 41.27 | -11.35 | 0.00 | 12.91 | 45.61 | -9.51 | 0.00 |
| 6h | 16.02 | 37.36 | -11.60 | 8.13 | 32.61 | 24.58 | -5.54 | 23.87 | 58.26 | 13.87 | 16.13 | 61.00 |
| 12h | 14.30 | 39.72 | -10.72 | 5.59 | 28.15 | 27.03 | -7.66 | 18.72 | 55.06 | 14.44 | 13.39 | 57.21 |
| 24h | 14.96 | 40.43 | -10.60 | 5.09 | 23.68 | 29.29 | -10.12 | 13.99 | 49.62 | 15.84 | 8.94 | 50.74 |
| 48h | 14.04 | 41.61 | -9.25 | 3.24 | 19.12 | 34.11 | -11.90 | 7.62 | 45.46 | 17.01 | 6.02 | 46.03 |
| 72h | 12.10 | 43.98 | -3.25 | 4.04 | 16.03 | 39.05 | -11.27 | 2.29 | 37.26 | 21.37 | 0.48 | 35.78 |
| Mean ΔE | 5.22 ± 1.87a | | | | 13.3±8.59ab | | | | 50.15±20.36A | | | |

Values are expressed as mean ± standard deviation (SD, n = 5). ΔE: total color difference calculated according to the CIE L*a*b* system. Different lowercase letters (a, b, c) indicate significant differences between groups at *p* < 0.05, while uppercase letters (A, B, C) indicate highly significant differences at *p* < 0.01, according to Tukey’s HSD test.

**Table S6.** Volatile metabolites (VMs) of ***Papiliotrema flavescens* RPT (*P. flavescens* RPT) under realistic storage condition**.

| **Classification** | **Formula** | **CAS NO.** | **Compounds** | **PF** | **NPF** | **log_2_ FC** | **p-value** | **adjusted p-value** |
| --- | --- | --- | --- | --- | --- | --- | --- | --- |
| Aliphatic | C12H22O3 | 63169-61-9 | 2-Methylpentanoic anhydride | 5.47 ± 1.84 | 13.92 ± 0.22 | -1.35 | 0.0014 | 0.013 |
| Aliphatic | C10H20O | 112-31-2 | Decanal | 33.02 ± 5.82 | 51.29 ± 4.8 | -0.64 | 0.0138 | 0.046 |
| Aliphatic | C9H18O | 124-19-6 | Nonanal | 62.69 ± 1.12 | 74.46 ± 2.65 | -0.25 | 0.0021 | 0.016 |
| Aliphatic | C12H26 | 17312-55-9 | 3,8-Dimethyl-decane | 11.6 ± 1.37 | 6.92 ± 0.84 | 0.75 | 0.0072 | 0.031 |
| Aliphatic | C8H18 | 563-16-6 | 3,3-Dimethyl-hexane | 18.28 ± 4.64 | 5.59 ± 2.4 | 1.71 | 0.0136 | 0.046 |
| Aliphatic | C17H34O2 | 112-39-0 | Hexadecanoic acid methyl ester | 7.32 ± 1.8 | 2.86 ± 0.05 | 1.36 | 0.0128 | 0.046 |
| Aliphatic | C7H14O | 110-43-0 | 2-Heptanone | 50.32 ± 12.87 | 4.06 ± 1.4 | 3.63 | 0.0035 | 0.020 |
| Aliphatic | C9H14O | 78-59-1 | Isophorone | 18.02 ± 1.83 | 11.9 ± 1.06 | 0.60 | 0.0074 | 0.031 |
| Aromatic | C8H8O3 | 119-36-8 | Methyl salicylate | 1213.33 ± 106.8 | 141.49 ± 6.03 | 3.10 | 0.0001 | 0.001 |
| Aromatic | C7H6O | 100-52-7 | Benzaldehyde | 23.03 ± 0.2 | 35.91 ± 0.84 | -0.64 | 0.0000 | 0.001 |
| Aromatic | C8H8O2 | 121-71-1 | 1-(3-Hydroxyphenyl)-ethanone | 7.1 ± 1.29 | 2.45 ± 0.23 | 1.53 | 0.0036 | 0.020 |
| Aromatic | C7H8 | 108-88-3 | Toluene | 20.33 ± 4.98 | 3.78 ± 0.05 | 2.43 | 0.0045 | 0.023 |
| Aromatic | C13H18O | 81561-77-5 | 1-(4-tert-Butylphenyl)propan-2-one | 31.81 ± 1.09 | 24.28 ± 2.03 | 0.39 | 0.0048 | 0.023 |
| Aromatic | C9H12O2 | 4179-19-5 | 3,5-Dimethoxytoluene | 6.49 ± 0.88 | 4.22 ± 0.22 | 0.62 | 0.0122 | 0.046 |
| Terpenoid | C10H12 | 1124-20-5 | p-Cymene | 35.36 ± 4.02 | 14.07 ± 2.03 | 1.33 | 0.0012 | 0.013 |
| Terpenoid | C10H18O | 106-25-2 | Nerol | 291.57 ± 22.42 | 390.41 ± 13.94 | -0.42 | 0.0029 | 0.020 |
| Terpenoid | C10H18O | 78-70-6 | Linalool | 2583.64 ± 191.64 | 1690.07 ± 164.69 | 0.61 | 0.0036 | 0.020 |
| Terpenoid | C13H18O | 1203-08-3 | 3,4-Dehydro-β-ionone | 113.66 ± 8.32 | 171.89 ± 11.68 | -0.60 | 0.0022 | 0.016 |
| Terpenoid | C11H16O2 | 17092-92-1 | Dihydroactinidiolide | 39.41 ± 2.51 | 29.04 ± 1.98 | 0.44 | 0.0049 | 0.023 |
| Terpenoid | C10H16 | 99-85-4 | γ-Terpinene | 42.58 ± 3.61 | 13.85 ± 0.37 | 1.62 | 0.0002 | 0.002 |
| Terpenoid | C10H16 | 13466-78-9 | 3-Carene | 145.71 ± 7.88 | 52.71 ± 4.82 | 1.47 | 0.0001 | 0.001 |
| Terpenoid | C10H16 | 5989-27-5 | D-Limonene | 409.04 ± 23.45 | 113.82 ± 10.61 | 1.85 | 0.0000 | 0.001 |
| Terpenoid | C10H18O | 10482-56-1 | α-Terpineol | 819.48 ± 7.04 | 647.84 ± 34.21 | 0.34 | 0.0010 | 0.013 |
| Terpenoid | C10H16 | 3779-61-1 | β-Ocimene | 82.47 ± 4.87 | 25.27 ± 2.75 | 1.71 | 0.0001 | 0.001 |
| Aliphatics |  |  |  | 206.73 ± 2.24 | 170.98 ± 2.08 | 0.274 |  |  |
| Aromatics |  |  |  | 1302.08 ± 83.01 | 212.14 ± 5.28 | 2.618 |  |  |
| Terpenoids |  |  |  | 4562.92 ± 167.93 | 3148.97 ± 171.80 | 0.535 |  |  |

Values are expressed as relative concentrations calculated by the internal standard method and presented as mean ± standard deviation (SD). VMs were considered significantly different between groups when both *p* < 0.05 and FDR-adjusted *q* < 0.05 were satisfied. Log₂ fold change (log₂FC) indicates the direction and magnitude of variation, with positive values representing up-regulation and negative values representing down-regulation in the inoculated group.

**Figure S1.** Relative abundance of dominant fungal phylum under different water activity conditions.

**Figure S2.** Heatmap of differential non-volatile metabolites (NVMs) in RPT samples stored under different water activity conditions.
